# Supplementary material for: Spatial constraints and stochastic seeding subvert microbial arms race
Source: PLoS Comput Biol. 2024 Jan 26;20(1):e1011807. doi: 10.1371/journal.pcbi.1011807 (PMC10849242; doi:10.1371/journal.pcbi.1011807)
Supplement: S1 Text — Fig A: Finite size effects are independent of κF. Fig B: Stochastic Seeding is a major factor in all starting conditions. Fig C: 64 generations leads to semi stable frequencies. Fig D: 64 generations leads to consistent final frequencies. Fig E: Finite size effects are independent of cell growth being limited by low pressure threshold. Fig F: Finite size effects are independent of cell growth being limited by medium pressure threshold. Fig G: Finite size effects are independent of cell growth being limited by high pressure threshold. Fig H: Finite size effects are independent of cell growth being limited by packing fraction. Fig I: Finite size effects are independent of cell growth choice being limited by carrying capacity. Fig J: Killing events prevent jamming in large systems. Fig K: Low Pressure Threshold growth leads to low inter-strain contact. (PDF) [file pcbi.1011807.s001.pdf]

Supporting information

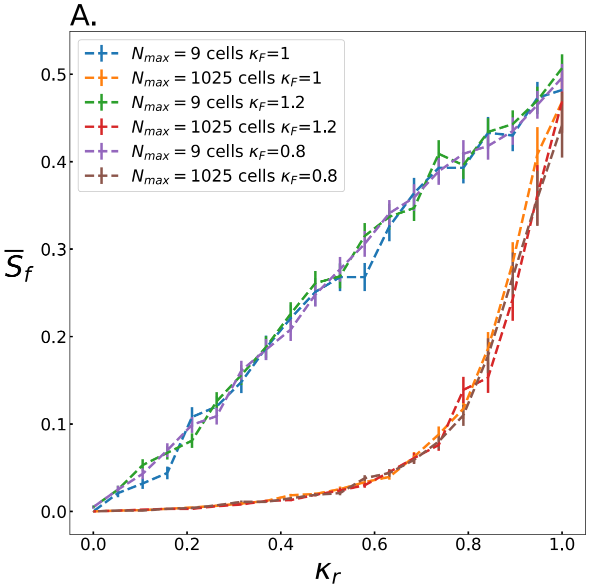

Figure A in S1 Text: Finite size effects are independent of κ_F

It is possible that the finite size effects observed were dependent on the fast killing strain's killing rate $\kappa_F = 1$. In order to discern if this was the case, we ran simulations were $\kappa_F = 0.8$ and $\kappa_F = 1.2$ in the $L = 3$ and $L = 31$ spaces and recorded $\overline{S}_f$ similar to Fig. 2 in the main text.

We found that changing κ_F had no effect in either the small or large environments Fig. A. The trend lines for all values of κ_F followed the same trajectory where the small environments produced a linear relationship between $\overline{S}_f$ and κ_r from $\kappa_r = 0$ to $\kappa_r = 1$ while the large environments led to no survival of the slow killing strain below $\kappa_r < 0.4$.

$$\gamma^* = \frac{\overline{S}_{f,s} - \overline{S}_{f,d}}{MAX(|\overline{S}_{f,s} - \overline{S}_{f,L}|, |\overline{S}_{f,s} - \overline{S}_{f,L}|)} \tag{1}$$

To demonstrate that the finite size effects observed in our study are not contingent on specific model choices used in our simulations, we conducted a thorough analysis of other potential model parameters. Initially, we identified the fundamental assumptions and choices underpinning our model. Subsequently, we modified our simulations to examine many possible combination of these factors.

Our model originally constrained cell growth when a global packing fraction reached a predefined threshold (equation 4 in the main text). However, alternative growth limitations exist. For instance, growth could be regulated by a numerical carrying capacity, limiting cell division but not expansion of an individual cell radius, once this capacity is maximized. This approach was explored by determining the maximal cell count that could fit into a given space without overlapping, using the smallest possible cell radius. We termed this growth limitation "carrying capacity", see Fig. I.

Another method to limit growth involves halting it for individual cells based on exerted pressure. This approach fosters more dynamic growth, allowing peripheral cells

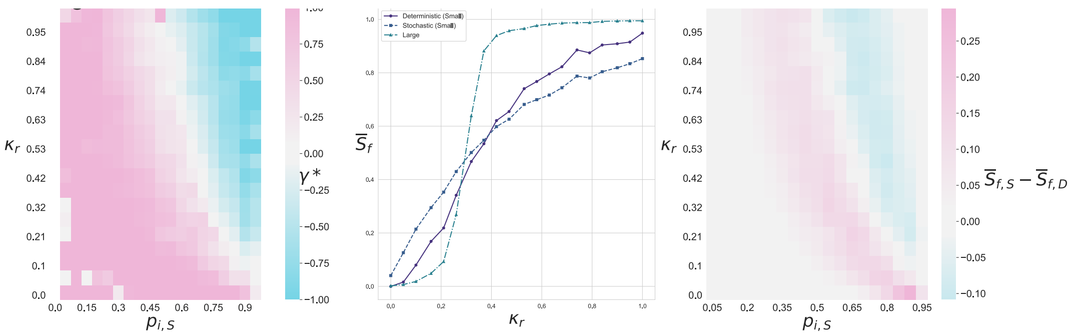

Figure B in S1 Text: Stochastic Seeding is a major factor in all starting conditions In the heatmaps above, $p_{i,S}$ is the initial proportion of the slow killing strain in the planktonic suspension, and κ_r is the ratio of the killing rate of the slow killing strain to the killing rate of the fast killing strain. $\overline{S}_f$ in the middle panel is the final proportion of the slow killing strain after 64 generations averaged over 1000 simulations for the small colony sizes and 30 for the large. In the left panel, we plot γ^* (see equation 1) on the z axis. Please note there are conditions where the calculation failed as the values were greater than 1 or less than -1. This occurred as the values on the edges where the denominator is very near zero. We replaced any value greater than 1 with 1, and less than -1 with -1.

in a cluster to expand more than those in densely packed areas [**?**]. We tested this by setting various pressure thresholds (1, 2.5, and 5) and observed that at $P_{thres} = 1$, the system was below the jamming point at the end of 64 generations, whereas at $P_{thres} = 5$, it surpassed the jamming threshold by the end of the simulation K. We termed this growth limitation "pressure threshold", see Fig. E-G.

Another key aspect of our model was the use of rigid boundaries (termed hard boundaries), where forces were applied via a harmonic potential to push cells inward should they reach the system edge. To assess the impact of other boundary conditions, we incorporated simulations with periodic boundaries, thereby negating any influence from boundary geometry. Note, whenever the combination of periodic bounds and growth being limited by packing fraction was tested, the maximum packing fraction was set to 0.84. Fig. E-I show simulations with periodic bounds in the left columns and hard boundaries in the right columns.

Furthermore, our initial model began with all cells at their minimum radius, representing an extreme scenario. To explore more conditions, we also simulated scenarios where cells started with random or maximum radii. Fig. E-I have "Min", "Max", and "Random" labels on the rows which mark the simulations' starting radii.

Lastly, in the original model, cells began with randomly placed centers, leading to significant initial overlap. To address this, we introduced a relaxation phase lasting one generation, during which cells could not grow or die but only move according to their harmonic interaction potentials. This adjustment ensured a minimal overlap when the competition begins while preserving the randomness of cell positions. All panels in Fig. E-I have a title which states that the relaxation period is True or False; True corresponds to the new approach with minimum starting overlap and False referees to random starting positions.

While we could test these choices in isolation, it is possible that some combinations of them would result in a shift in the effects reported in the study. Thus, Fig. E-I, show all possible combinations. We find that none of the combinations of model choices affected the general trends described in this paper. The slow killing strain always follows a linear trend from $\overline{S}_f = 0$ to $\overline{S}_f = 1$ in small spaces, and $\overline{S}_f$ is always less in

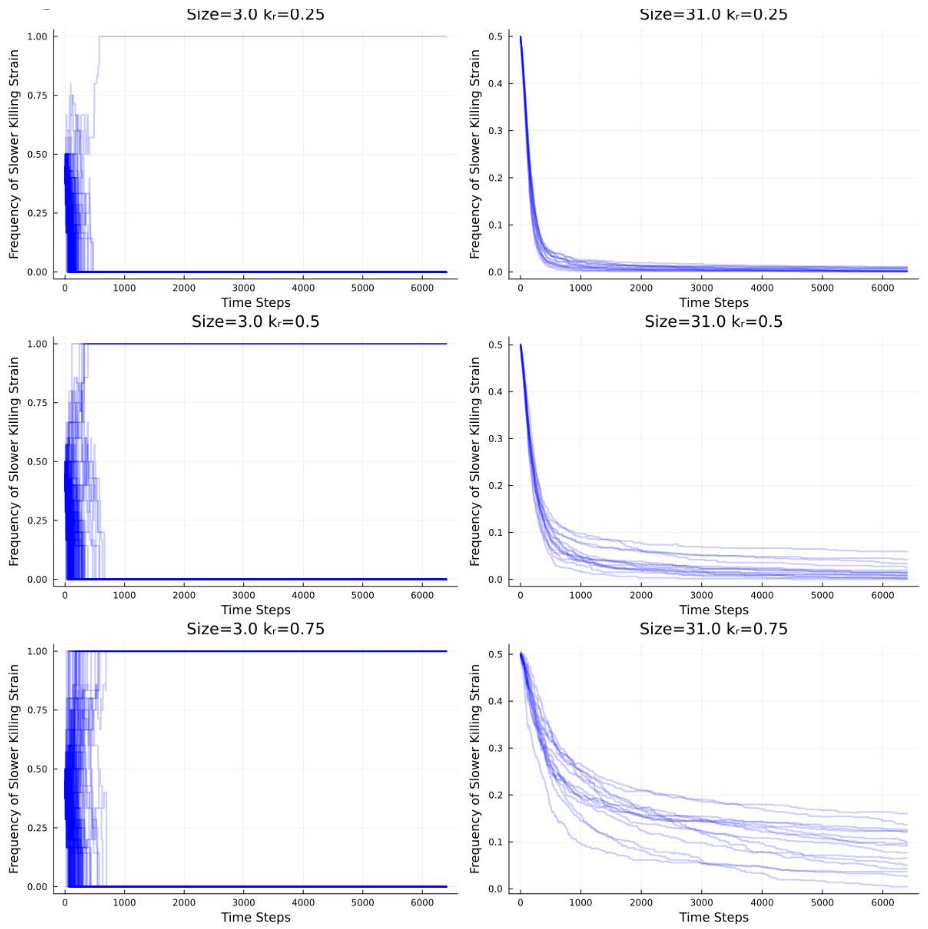

Figure C in S1 Text: 64 generations leads to semi stable frequencies The frequency of the slow killing strain is plotted versus time steps; every 100 time steps is a generation as this is the average time taken for one cell to divide. All panels show 15 trends, and the parameters for these simulations match those in the main text.

large spaces.

The low pressure threshold ($P_{thres} = 1$) results in the difference between the large and small colony space trends being smaller Fig. E; however, we found this is simply due growth stopping in large populations before the environment is densely packed, which prevents continued competition and either strain from overtaking the space. Thus, slow killing cells survive as they stop contacting fast killing cells Fig. K.

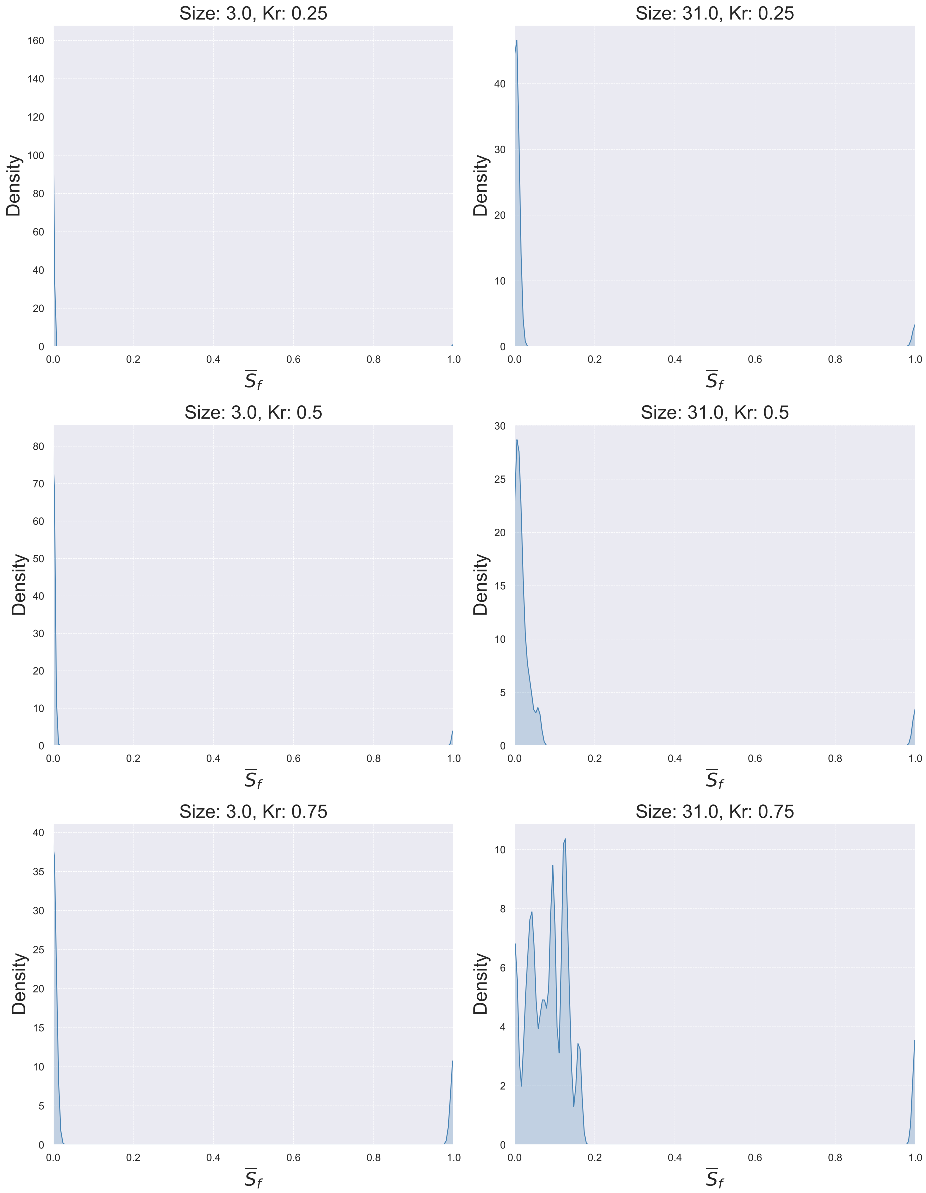

Figure D in S1 Text: 64 generations leads to consistent final frequencies Here we show the probability density functions of the final proportions of the slow killing strain taken from the frequency over time examples Fig. E.

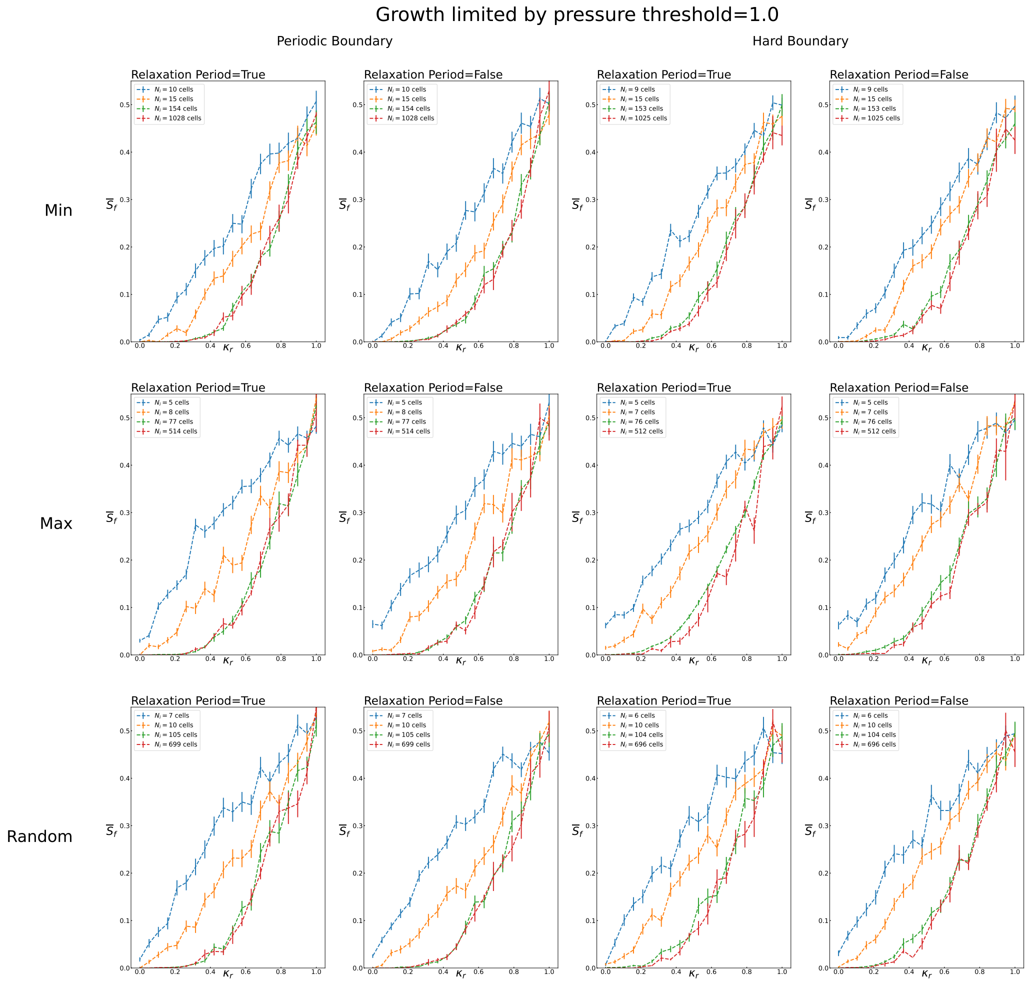

Figure E in S1 Text: Finite size effects are independent of cell growth being limited by low pressure threshold Above we show all parameter combinations of simulations where growth is limited by pressure on individual cells where the threshold is set to 1. We see that the effect described in our manuscript is robust to how the simulation is performed; the small colonies always result in a significant increase in frequency.

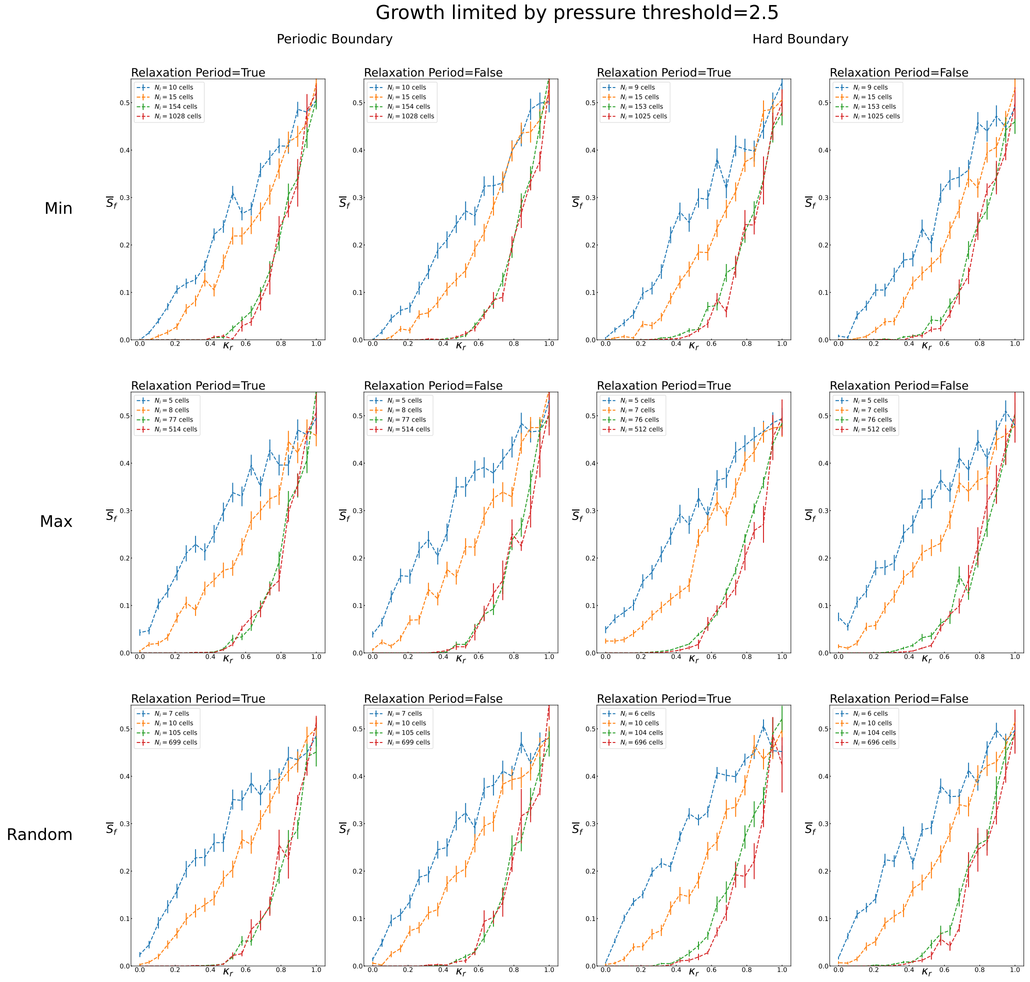

Figure F in S1 Text: Finite size effects are independent of cell growth being limited by medium pressure threshold Above we show all parameter combinations of simulations where growth is limited by pressure on individual cells where the threshold is set to 2.5. We see that the effect described in our manuscript is robust to how the simulation is performed; the small colonies always result in a significant increase in frequency.

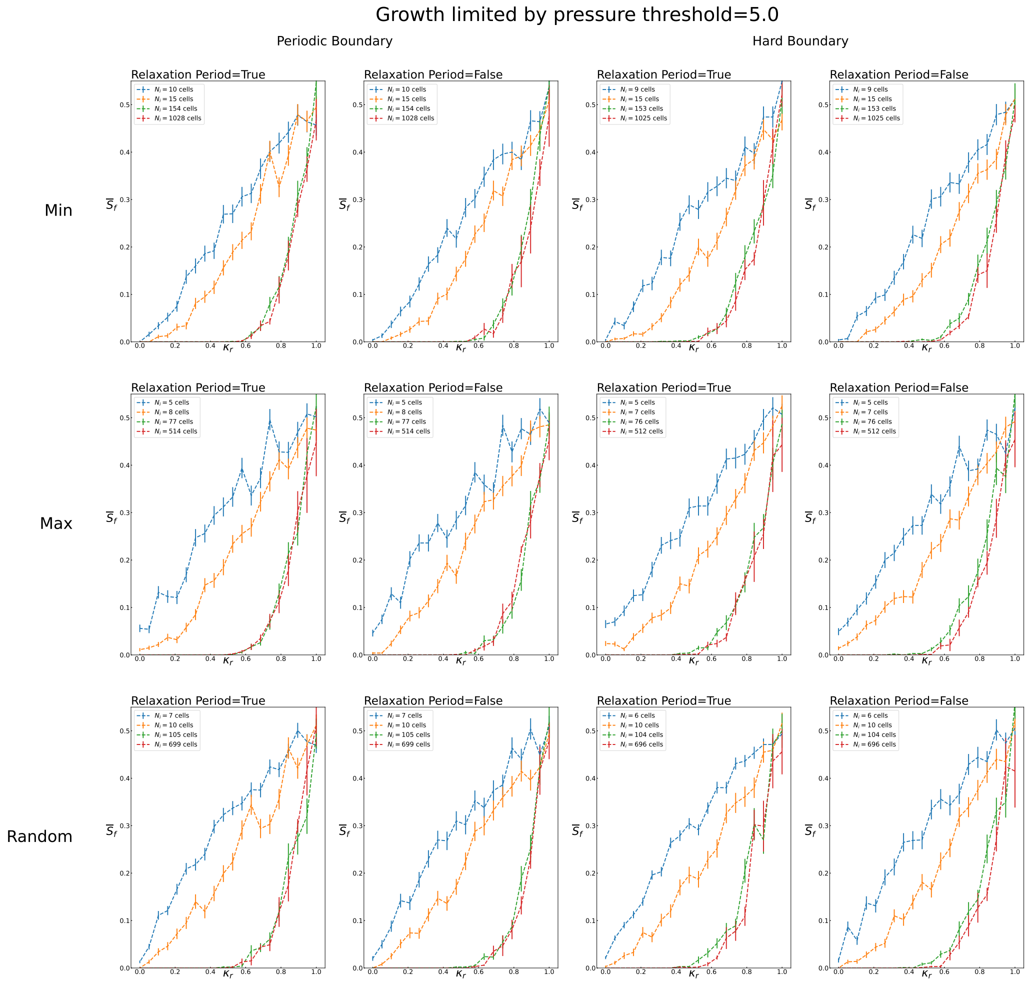

Figure G in S1 Text: Finite size effects are independent of cell growth being limited by high pressure threshold Above we show all parameter combinations of simulations where growth is limited by pressure on individual cells where the threshold is set to 5. We see that the effect described in our manuscript is robust to how the simulation is performed; the small colonies always result in a significant increase in frequency.

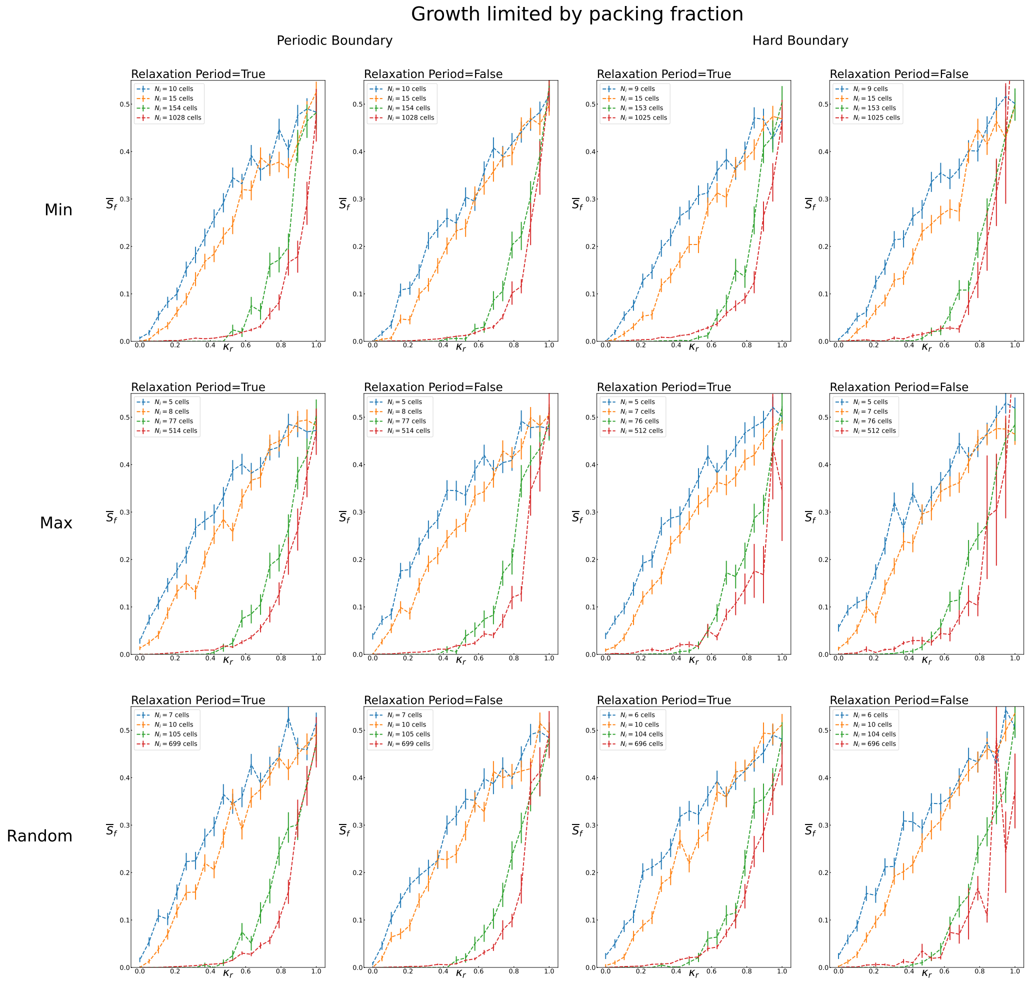

Figure H in S1 Text: Finite size effects are independent of cell growth being limited by packing fraction Above we show all parameter combinations of simulations where growth is limited by the packing fraction as described in our original manuscript. We see that the effect is robust to how the simulation is performed; the small colonies always result in a significant increase in frequency.

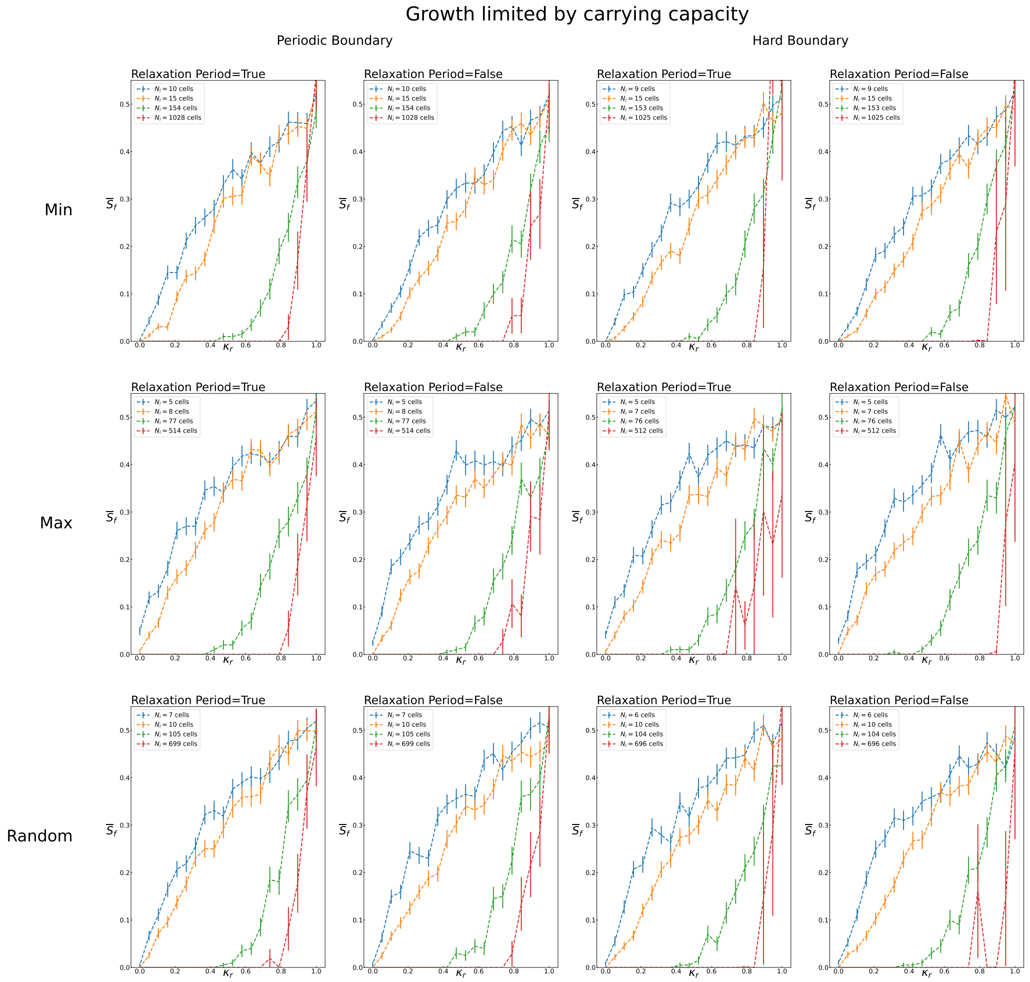

Figure I in S1 Text: Finite size effects are independent of cell growth choice being limited by carrying capacity Above we show all parameter combinations of simulations where growth is not limited but rather a maximum number of cells for a given space is set. We see that the effect is robust to how the simulation is performed; the small colonies always result in a significant increase in frequency.

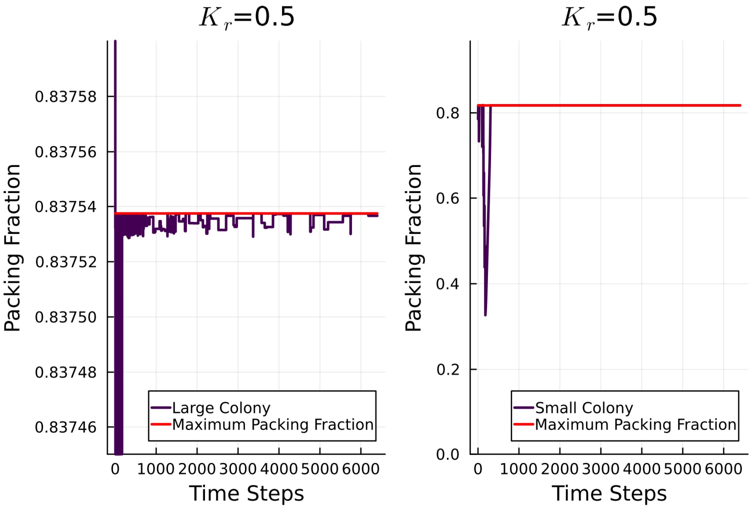

Figure J in S1 Text: Killing events prevent jamming in large systems Here the packing fraction of the whole colony is plotted against the time steps of the simulation where 100 time steps is a generation. The packing fraction of the large colony (1025 cells) fluctuates throughout our entire simulations, and never remains at the jamming transition for long before decreasing. Small colonies (9 cells) experience large fluctuations in packing fraction, until one of the two strains goes extinct; the packing fraction then stabilizes at the jamming transition. Thus, in each of these cases, the impact of stresses on growth rates is not relevant for the competitive dynamics between the two strains.

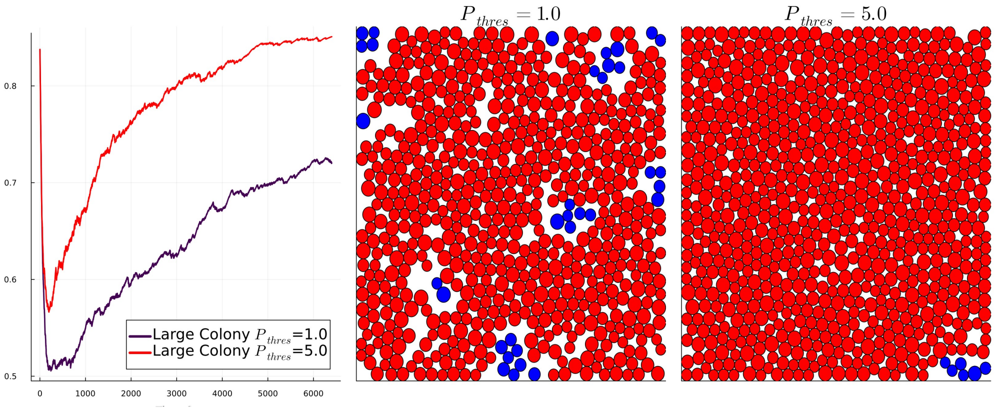

Figure K in S1 Text: Low Pressure Threshold growth leads to low inter-strain contact The left panel plots the packing fraction against time. We see packing fraction drops rapidly and then slowly rises over time. However, at the end of the simulation it is far below the jamming threshold for the low pressure threshold and above it for the higher threshold. This leads the low threshold simulations to have large gaps between cells of opposing strains while the high pressure simulations allow the fast killing strain to overtake more space.The middle and right panels show visualizations of our simulations with the low and high threshold pressures after 64 generations.
