# Supplementary material for: Spatial constraints and stochastic seeding subvert microbial arms race
Source: PLoS Comput Biol. 2024 Jan 26;20(1):e1011807. doi: 10.1371/journal.pcbi.1011807 (PMC10849242; doi:10.1371/journal.pcbi.1011807)
Supplement: Attachment — Submitted filename: Copeland et al response letter.pdf [file pcbi.1011807.s002.pdf]

November 17, 2023

To whom it is concerned,

We were delighted by the overall positive reports from the referees. Referee2 described the paper as “interesting” and said that it “fulfills the PLoS Computational Biology requirements of originality, innovation, and importance to researchers in the field.” Reviewer 3 also called the paper “interesting” and stated that “The main question, whether small population sizes may alter the outcome of antagonism between such strains, is an important one.” Though Referee 1 had a mixed review, they did not question the validity. Reviewer 1 thought that the primary results were already known, believing them to be similar to the fact that “in small populations selection is less efficient.”

We will address all referee comments in detail below, and will briefly summarize the major changes made here. In response to Referee 1’s questions about the novelty of our approach compared to the non-spatial Wright-Fisher model, we now explicitly show that spatial effects substantially change the outcome of competitions. More importantly, we exactly quantify the impact of stochastic seeding, implicating it as the primary cause of many of the effects we describe; crucially, these seeding effects have not been described before, especially in the context of microbial warfare. Further, in response to questions about our simulation methodology from Reviewers 2 and 3, we implemented 60 new approaches to performing these simulations, and performed over 5 million new simulations. We find that for any reasonable approach, including all of the approaches suggested by reviewers, we obtain the same qualitative results. Finally, in response to Reviewers 2 and 3 we performed a crucial control experiment and competed our two antagonistic strains against each other in larger TEM grids.

We believe that addressing all referee comments significantly improved the manuscript, and thank all three reviewers for their useful questions and comments. Please do not hesitate to reach out if any clarification is necessary.

Sincerely,
Raymond Copeland
Peter J. Yunker

Reviewer #1:

Reponse: Thank you for your comments. We believe that addressing them has made the manuscript clearer. We hope the changes we have made to the manuscript address your concerns.

The paper examines the interplay of genetic drift and natural selection in a certain semi-realistic model of spatial growth. The basic result is that in small populations selection is less efficient. Overall, I don't find the results to be novel enough for Comp Bio, but the manuscript could go into PLoS ONE.

Response: We believe there has been a lack of clarity, and apologize for that. However, we also think you raised an important phenomenon for us to compare to, and believe that our manuscript has greatly improved by clarifying this topic.

There certainly are similarities between the phenomena described in our manuscript and natural selection/drift. Natural selection is more effective in large populations than in small populations, just as we find that contact killing advantages are more valuable in large populations than in small populations. In both systems, the difference between large and small populations comes from the importance of stochastic fluctuations. However, the source of stochastic fluctuations is different between these two scenarios.

To that end, the effects we report here primarily come from the fluctuations in the initial proportions of each strain while picking from the same planktonic suspension. This approach is crucial to replicating the random attachment of planktonic bacteria onto a surface. Specifically, the small populations allow for a broader range of initial conditions (Figure 4A and C); as these competitions always end with one strain being eradicated, this wide range of initial conditions allows the slower killing strain to win often enough to survive when seeded from more planktonic conditions (Figure 4B and D). However, there are of course more sources of randomness than initial seeding, and your comments inspired us to expand our analysis of observed stochastic fluctuations and directly compare our model to the Wright Fisher model. We hope our specific responses below meet that end.

To more rigorously quantify the importance of seeding, we compared the mean outcome of simulations with fixed, i.e., deterministic, initial conditions to the mean outcome of simulations with stochastically set initial conditions (comparing simulations of each kind with the same mean initial conditions).

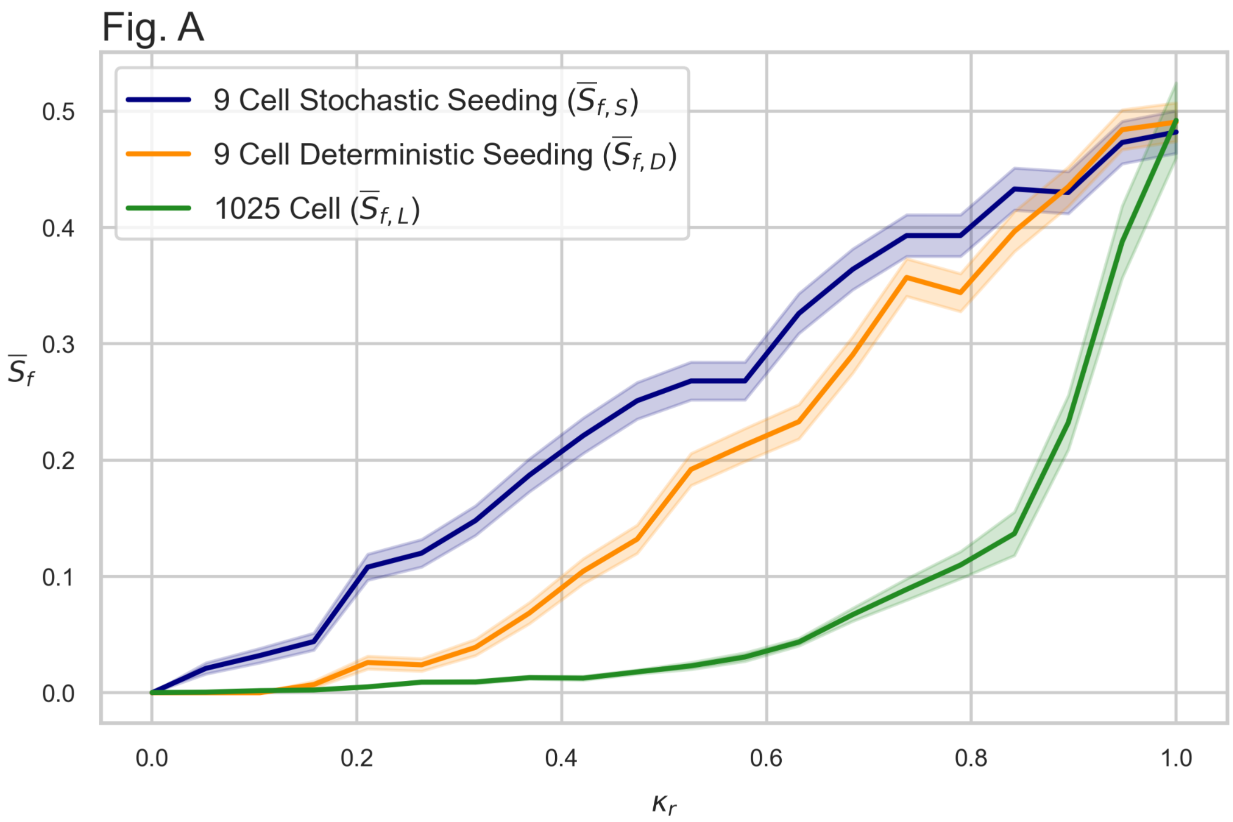

Fig. A. S_f is the final proportion of the slow killing strain, and k_r is the killing rate ratio of the slow killing strain to the fast killing strain. Stochastic seeding means that the initial abundance of the slow killing strain was randomly selected from a binomial distribution with equal chance of either strain and the total cell count = 9. Deterministic seeding with an equal proportion is impossible for 9 total cells, so we averaged the trends where the initial abundance of the slow killing strain is held at 4 and 5 cells out of 9. There are 20 data points in this trend line; each point is the result of 1000 simulations. These two lines describe simulated “small colony” sizes. The 1025 cell simulations are also stochastically seeded; the initial proportion is expected to be 0.5 +- 0.016. There are 20 data points in this

trend line, and each is the average of 50 simulations. This line describes a simulated “large colony”. The error bars represent standard error.

Indeed, we see that stochastic seeding substantially increases the average slow killing strain’s population size as compared to deterministic seeding (with all other sources of stochasticity present). Below, we quantify how much of the difference between small and large spaces comes from stochastic seeding, versus other stochastic factors . We define impact (ɣ) as the ratio of the difference between (stochastic-deterministic) and (stochastic-large cell number). We find that the proportion of the effect due to seeding is greatest at low k_r. Further, stochastic seeding is responsible for the majority of the difference between large and small spaces when k_r < 0.5.

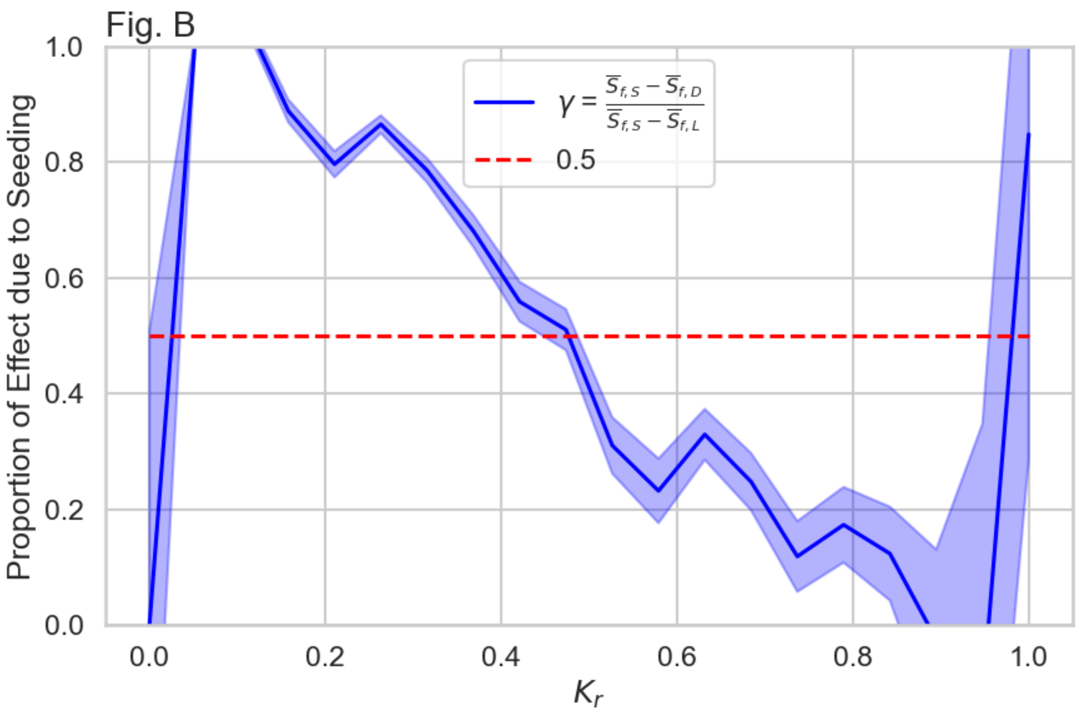

Fig. B K_r is the same killing ratio shown in the previous figure (Fig. A), and ɣ is the difference between the stochastic and deterministic small colony seeding trends divided by the difference between the stochastic, small colony and the large colony trends. This quantifies how much of the “total finite size effect,” i.e., the difference between large and small simulations, is a result of stochastic seeding.

Georgia Institute of Technology

Further, we used the data from Figure 3 of the manuscript to quantify the importance of seeding (ɣ) for invasion as well.

Fig. C

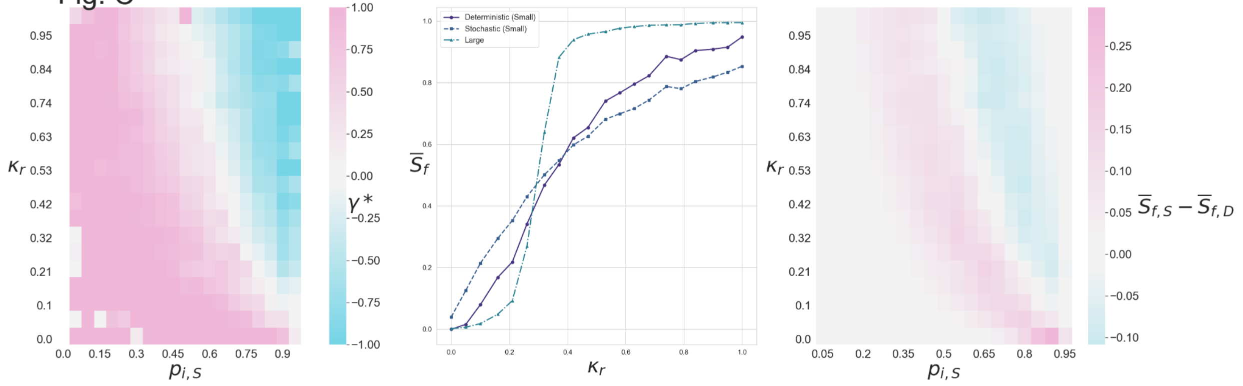

Fig. C In the heatmaps above (Fig. C) , P_i,s is the initial proportion of the slow killing strain in the planktonic suspension, and K_r is the ratio of the killing rate of the slow killing strain to the killing rate of the fast killing strain. S_f in the middle panel is the final proportion of the slow killing strain after 64 generations averaged over 1000 simulations for the small colony sizes and 30 for the large. In the left panel, we plot ɣ* on the z axis. It is calculated by taking the ratio of the final fraction of the slow killing strain in stochastic seeding conditions minus the final fraction of the slow killing strain in deterministic seeding conditions to the larger of:

1. the final fraction of the slow killing strain in stochastic seeding conditions in small populations and the final fraction of the slow killing strain in large populations
2. The final fraction of the slow killing strain in deterministic seeding conditions and the final fraction of the slow killing strain in large populations.

Mathematically, this is calculated as:

$$\gamma^* = s_{f,s} - s_{f,d} / MAX(|s_{f,s} - s_{f,L}|,|s_{f,d} - s_{f,L}|)$$

This is similar to gamma introduced above. The sole difference is that the denominator is now the absolute value of the largest difference of either option 1 or 2. We made this change as sometimes the stochastic seeding does not aid the slow killing strain but actually impedes it (See Fig C middle panel). While our original manuscript reported that

finite size effects aid the fast killing strain when at a numeric disadvantage, here we can see that a major factor behind that observation was stochastic seeding effects.

We see that seeding has a major impact across these conditions, and in fact, it is greater than 0.5 or less than -0.5 in 90% of conditions. However, this proportion does not reflect how large the effect is. Therefore, we show the difference between stochastic and deterministic seeding in small populations in the right panel of Fig. C. We see that as before, the importance of seeding increases as k_r decreases and can lead to a total frequency increase of 30%. These graphs will go in the supplementary material.

Further, we are altering our Figures 2 to 5 in the body of the manuscript to directly address stochastic vs deterministic seeding. We will address these changes in response to your next comment.

Thank you for pointing out this comparison; we think by explicitly commenting and quantifying seeding as a major driver of these effects, the manuscript is clearer, and much improved.

Main suggestions:
1. I would supplement each figure with the corresponding plot of the Wright-Fisher model with the matched selection coefficient and population size. This way one could see how much of the effect is "generic" (i.e. the same as in the WFM) and how much is due to the idiosyncrasies of the model including the effect of space and growth dynamics.

Response: Thank you for this suggestion. Unfortunately, matching killing rates and selection coefficients is non-trivial, especially considering that both strains grow at equal rates. Nonetheless, we appreciate this suggestion and agree that comparing to the Wright-Fisher Model would provide useful context for our results. We thus ran the Wright Fisher model for a variety of fitness values (64 generations each where the high fitness strain's fitness was set = 1, and the frequency of each initially is 0.5)

Fig. D

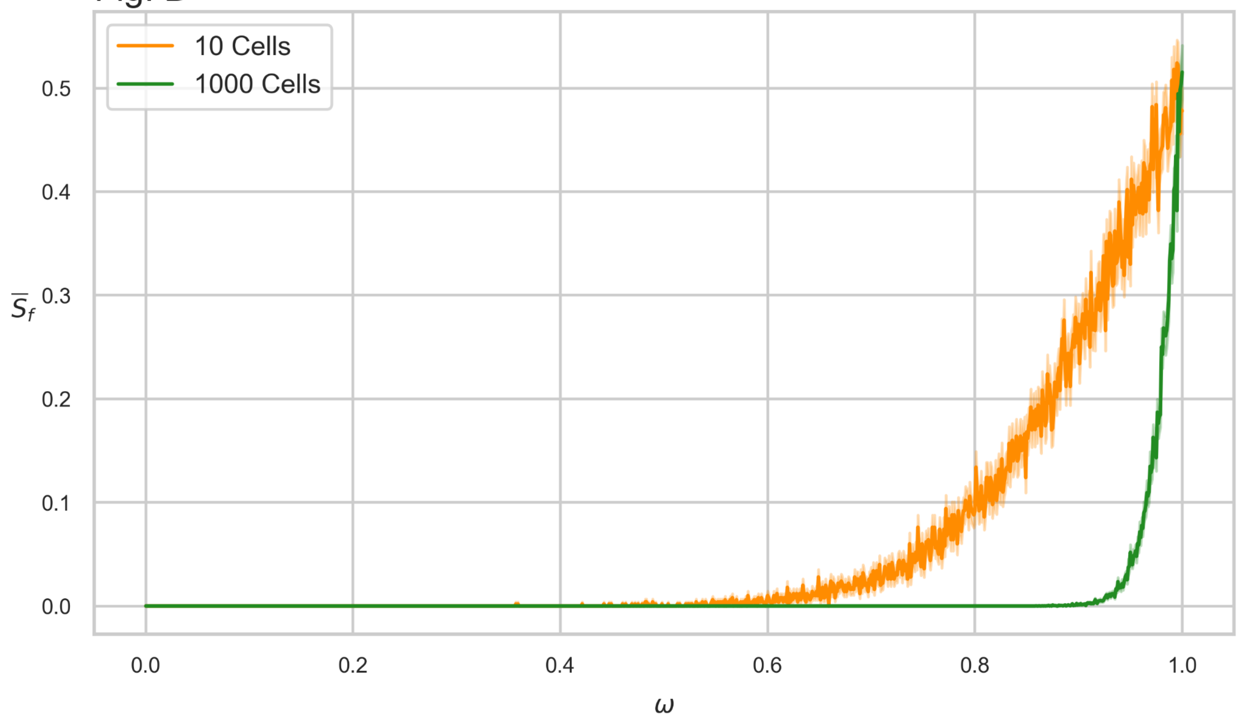

Fig. D S_f is the final proportion of the low fitness strain, averaged across 30 simulations. The trend line has 1000 data points, representing the means for different fitness values for the low fitness, ω. Each simulation was run for 64 generations.

This graph (Fig. D) shows the average final frequency of the less fit strain as a function of fitness with standard error filled in around the trend. We can see that drift in the Wright Fisher model also leads to a greater proportion of the less fit strain surviving, but the effect is less pronounced and non-linear. Unlike in our contact killing simulations, there is not survival in the small space for all conditions as our model shows in figure 2. Further, the comparison of fitness to k_r, or the comparison of s_f in the "large" cell WF simulations to our model is non-trivial and potentially problematic.

The differences between the two models comes from both seeding and spatial effects. The spatial effects are highly pronounced in the large space where phase separation drives a semi-stable long time frequency as seen in the Fig E. below, which plots the frequency of the slow killing strain as a function of time:

Fig. E

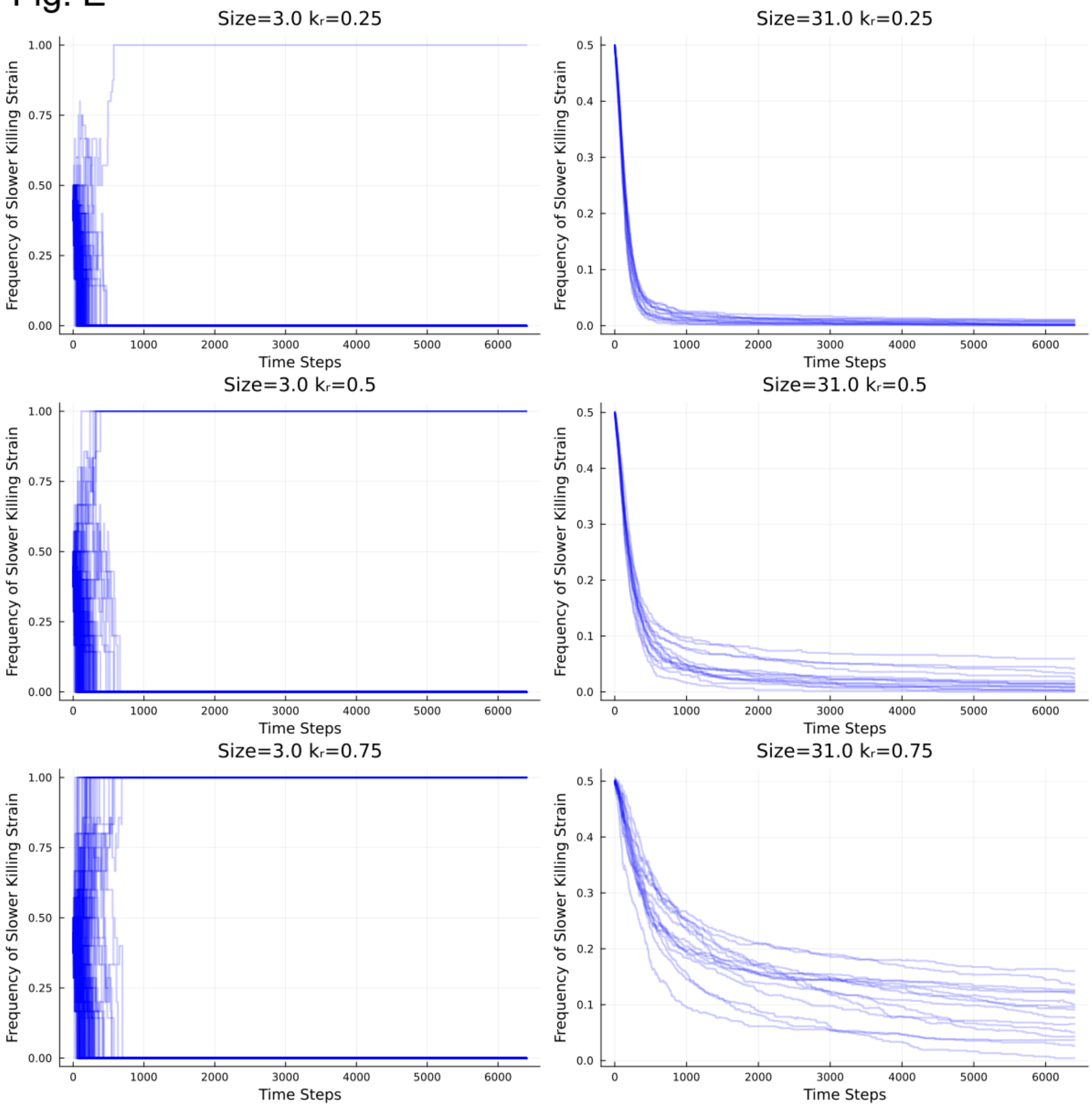

Fig. E The frequency of the slow killing strain is plotted versus time steps; every 100 time steps is a generation as this is the average time taken for one cell to divide. All panels show 15 trends. Size 3 and 31 correspond to 9 and 1025 cell simulations respectively.

And here are frequencies of the slow killing strain over time graphs of the WF model for large populations in fig F.

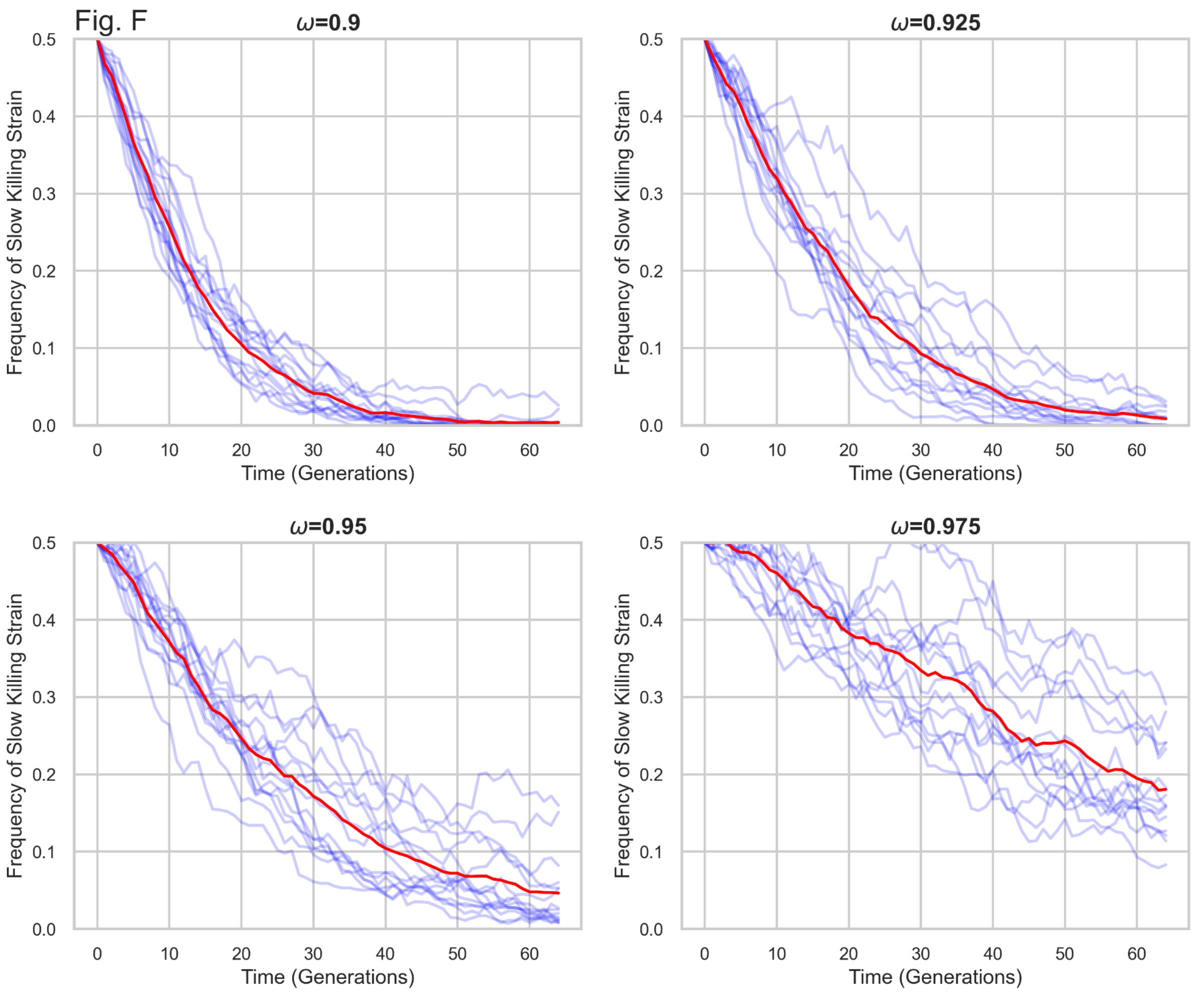

Fig. F The frequency of the low fitness strain is plotted against time, in generations, on the horizontal axis. The fitness value of the low fitness strain is shown in the title of each panel, and each panel shows 15 replicate simulations in blue with the mean shown in red.

We see here that while our simulations reach a ~stable state within 64 generations, the WF does not, so the difference between the large and small trends observed in the sf v omega

graph is only due to the time constraint while spatial effects in our simulations enforce a robust difference. The stability in our model comes from phase separation of the two strains into patches of one type and the other (Steinbach Gabi, Crisan Cristian, Ng Siu Lung, Hammer Brian K. and Yunker Peter J. (2020) Accumulation of dead cells from contact killing facilitates coexistence in bacterial biofilmsJ. R. Soc. Interface.172020048620200486) (Ghosh, Pushpita, et al. "Mechanically-driven phase separation in a growing bacterial colony." Proceedings of the National Academy of Sciences, vol. 112, no. 17, 2015, https://doi.org/10.1073/pnas.1504948112). This minimizes the interactions between the strains and will go away given infinite time, but as shown above, they are very stable given any relevant time frame. Examples of such interfaces in larger systems in our simulations are shown below in Fig. G.

Fig. G

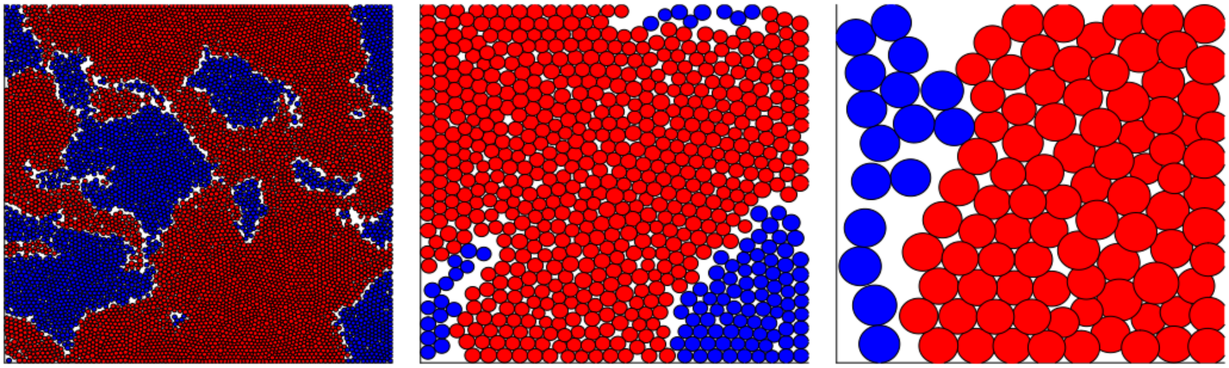

Fig. G Each panel shows a visualization of our simulations when killing rates are similar after 40 generations. The panels show 10,000, 100, and 100 cells respectively.

Thank you again for this suggestion; it motivated us to perform the analysis of the importance of stochastic fluctuations in small populations with identical initial conditions described above, which we think improved and clarified the manuscript. We added many of the figures in the response letter into the manuscript. Specifically, figures A and B (from the response letter) were added to figure 2 in the manuscript, figure G was added to figure 1, and figures C and E were added to the SI.

2. It would be good to see how frequencies and population size change during the 64

generations at least for some of the conditions.

Response: Thank you for this suggestion. We have added a figure to the supplemental information and also show it here.

Fig. E

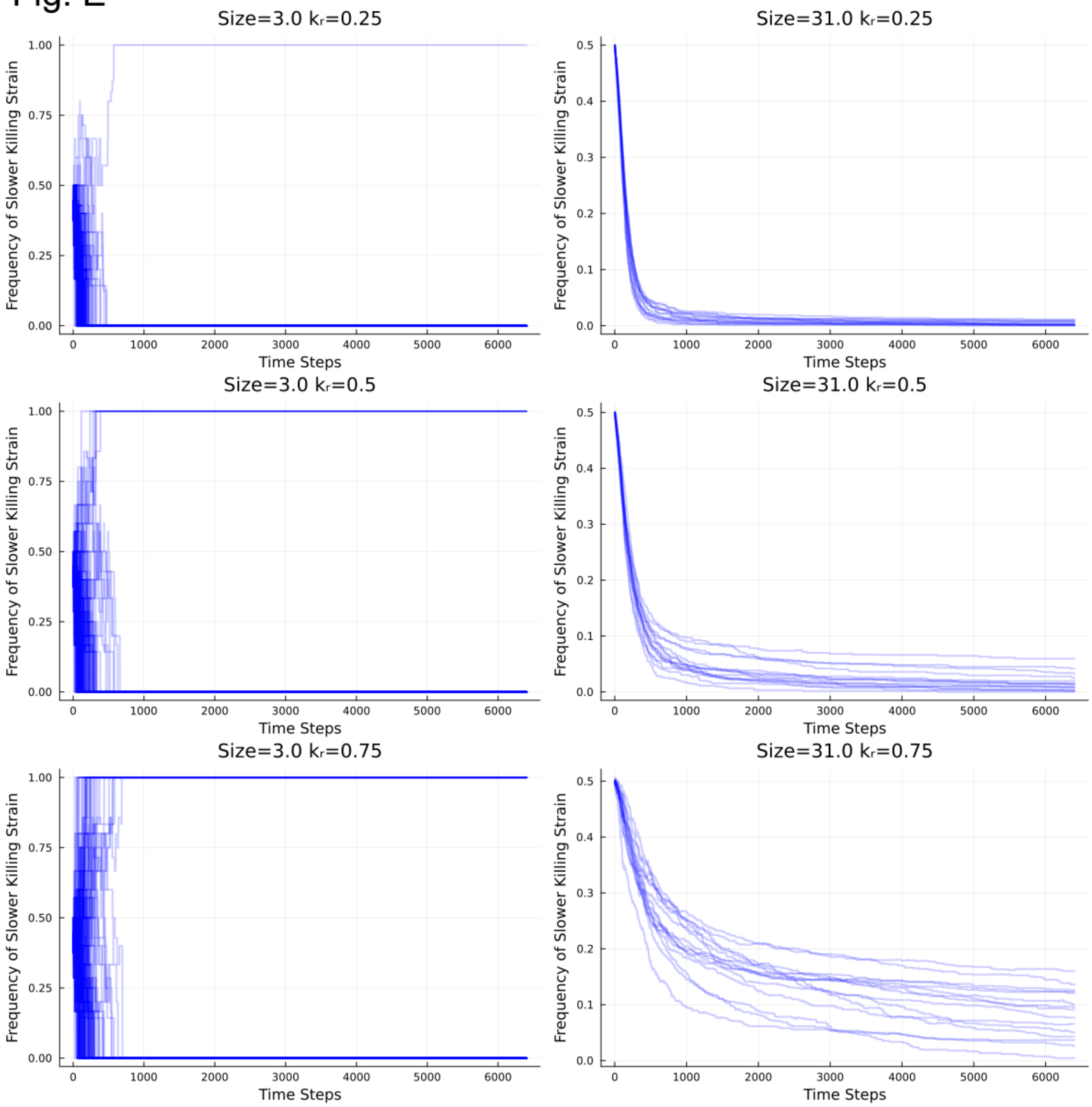

Fig. E The frequency of the slow killing strain is plotted versus time steps; every 100 time steps is a generation as this is the average time taken for one cell to divide. All panels show 15 trends. Size 3 and 31 correspond to 9 and 1025 cell simulations respectively.

3. It would be useful to see the distribution of frequencies in the final populations including the number of populations reaching fixation.

Response: We agree that it would be beneficial for readers to see this data so as to know whether the finite size effect reported is fixed or not. There is some difficulty in displaying these data as we could report over 200 histograms in Fig. 2 alone, for example. However, we added the figure below to the supplementary information with some representative examples.

Fig. H

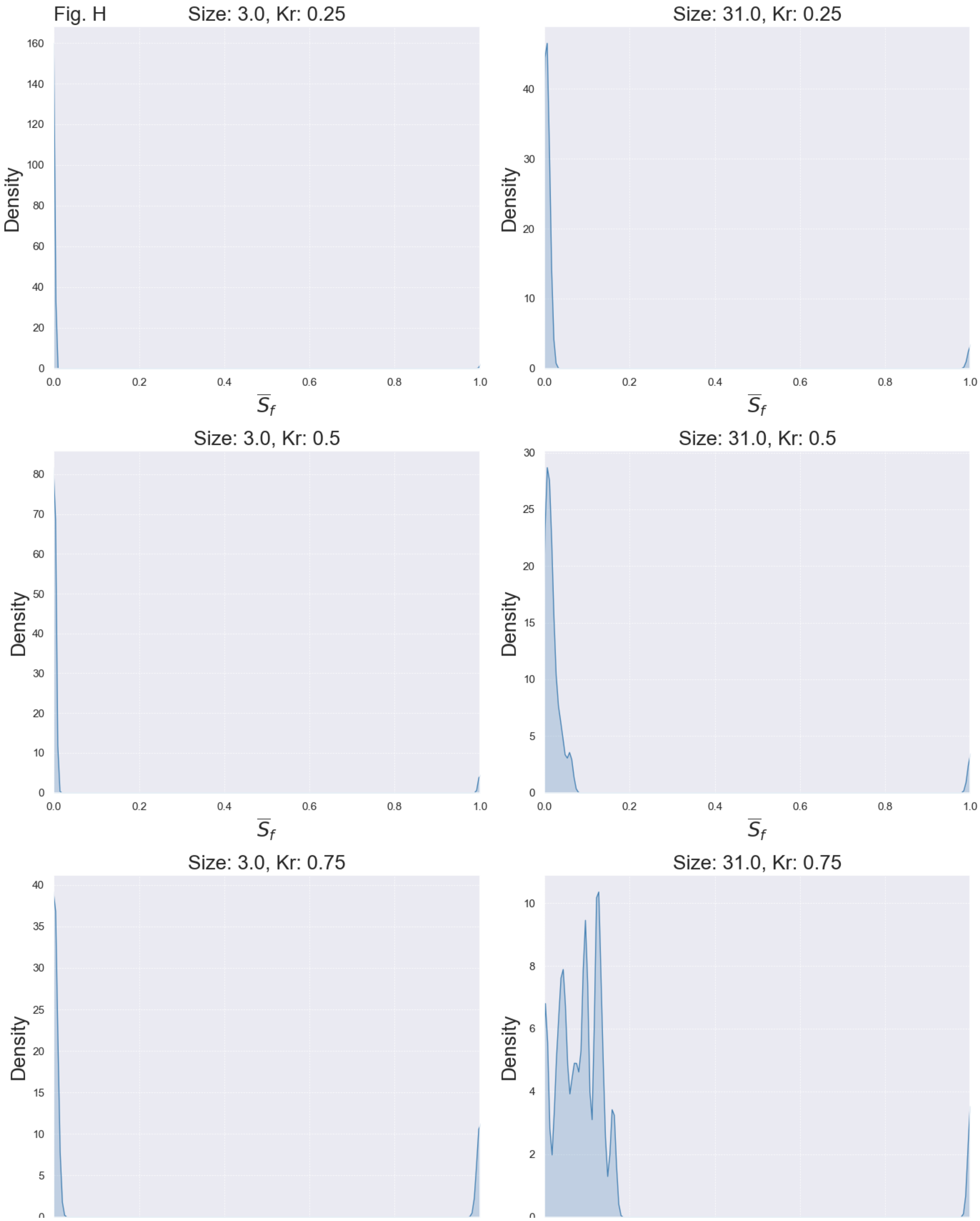
Size: 3.0, Kr: 0.25
Size: 31.0, Kr: 0.25
Size: 3.0, Kr: 0.5
Size: 31.0, Kr: 0.5
Size: 3.0, Kr: 0.75
Size: 31.0, Kr: 0.75
Density
$\overline{S}_f$

Fig. H Here we show the probability density functions of the final proportions of the slow killing strain taken from the frequency over time examples shown above.

Minor:
I couldn't find where the initial population size in each of the simulations is stated.

Response: The initial population size is stated in the "Simulation Methods" section. Please note, in the revised manuscript, we now explore a broader range of simulation parameters and model choices. The initial population size for the simulations presented in the main text remains in the "Simulation Methods" section, but details on other simulation approaches can be found in both the methods section and in the SI.

Reviewer #2: This is an interesting study. In it, the authors study the influence of size constraints and initial conditions on competition between two strains of bacteria that kill each other at different rates upon contact, which arises in many cases in microbiology. To my knowledge a similar study has not been performed before and this paper therefore fulfills the PLoS Computational Biology requirements of originality, innovation, and importance to researchers in the field. It therefore has potential to be publishable. However, there are several concerns relating to the methodology, data analysis, and interpretation of the results that need to be properly addressed before the manuscript can be published.

Response: Thank you for your kind words and insightful comments. We believe that addressing your comments improved the manuscript.

— A serious concern relates to how mechanical stresses are incorporated in the model. As I understand it (although the description was very unclear), cell growth stops if the global packing fraction exceeds a threshold. But it is well known that the stress can vary greatly spatially in a close packed colony. The stresses go to zero at the edges and compressive stresses build up near the center, which is required from mechanical equilibrium. This spatial variation in stresses which can have important implications for e.g. spatial variations in growth and growth rates is not incorporated at all, which is very concerning.

Response: Thank you for your comment. A lot of important and interesting work has been done on this topic in recent years; we agree that it is important to address. Below we will

first discuss the role of stress in our simulations, and then will more generally explore the impact (or more precisely, the lack of impact) of many different modeling decisions. To briefly summarize, until one strain goes extinct, killing keeps the packing fraction below the jamming point, making stress-limited growth irrelevant for the outcomes of these deadly competitions.

Our simulation approach was inspired by Gniewek, et al., Biomechanical Feedback Strengthens Jammed Cellular Packings, PRL 20119. There, the authors show that there is (effectively) no pressure in the system below the jamming threshold, an observation consistent with decades of work on jamming in non-living systems (O'hern, C. S., Silbert, L. E., Liu, A. J., & Nagel, S. R. (2003). Jamming at zero temperature and zero applied stress: The epitome of disorder. Physical Review E, 68(1), 011306.). Thus, any simulations that focus on the behavior of microbes above the jamming point must account for the impact of stress on individual cells.

However, as our system approaches the jamming transition, cells begin to contact each other and, thus, kill each other. As a result, the packing fraction drops. Our simulations only pass the jamming threshold when one of the strains has gone extinct. An example plot of the packing fraction vs. time is below.

Fig. I

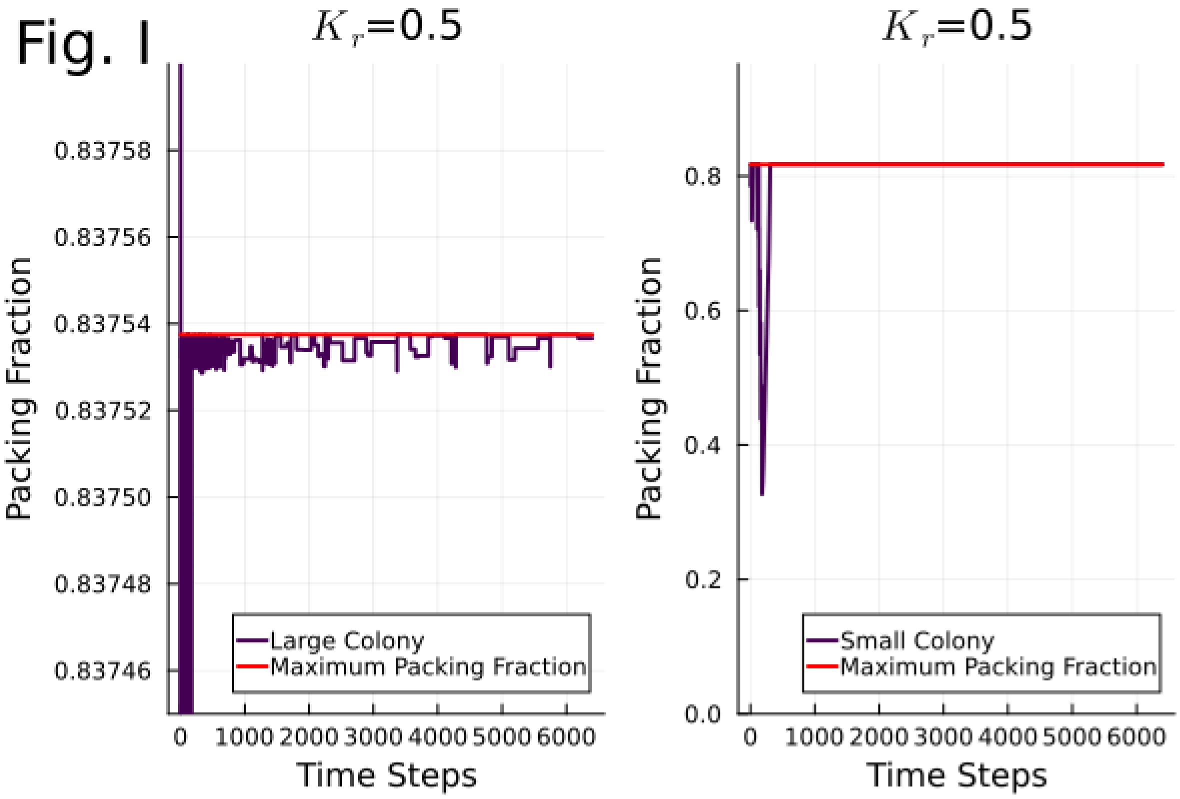

Fig. I Here the packing fraction of the whole colony is plotted against the time steps of the simulation where 100 time steps is a generation. The packing fraction of the large colony (1025 cells) fluctuates throughout our entire simulations, and never remains at the jamming transition for long before decreasing. Small colonies experience large fluctuations in packing fraction, until one of the two strains goes extinct; the packing fraction then stabilizes at the jamming transition. Thus, in each of these cases, the impact of stresses on growth rates is not relevant for the competitive dynamics between the two strains.

Though local stresses and their impact on growth rates thus should not impact our competitive outcomes, we sought to test this idea directly. We therefore redesigned our simulations to stop growth of individual cells should their pressure exceed a threshold value. Pressure is calculated for each cell by summing the magnitude of forces on it and dividing this quantity by the cell's circumference (Gniewek, et al., Biomechanical Feedback Strengthens Jammed Cellular Packings, PRL 20119). Note, stopping growth upon reaching a pressure threshold is an extreme case; typically growth is modeled as slowing when a threshold is reached, not completely stopping. We varied this value between 1, 2.5, and 5.0,

values that represent low, medium, and high pressures. We will add more context to these values below. The medium and high pressure thresholds produced results nearly identical to those in our manuscript:

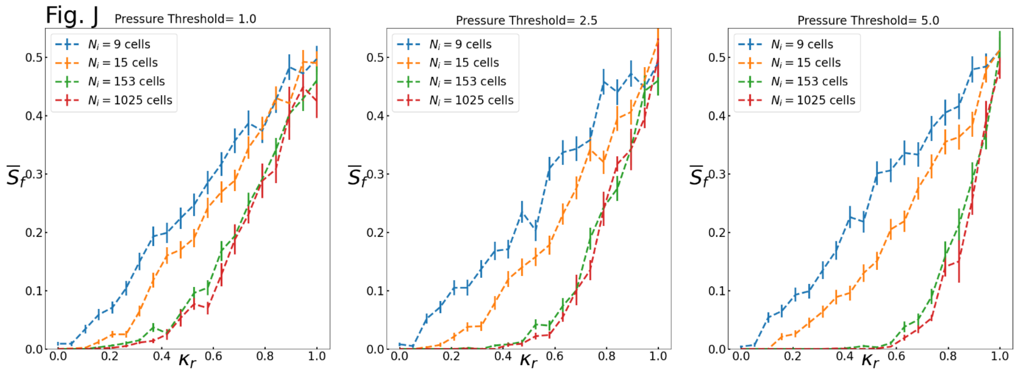

Fig. J Above, each panel plots the final proportion of the slow killing strain against the killing ratio for different colony sizes for simulations where growth stops when P = 1 (first panel), P=2.5 second panel, and P = 5.0 (third panel). The rest of the parameters for these simulations match those in the original manuscript.

In particular, the smallest populations studied are nearly identical to those reported in Figure 2 of our main text, and the slow killing strain survives at higher mean abundances in small populations than in large populations. The primary difference is the large populations; the relative abundance of the slow killing strain is higher in the simulations with the lowest pressure thresholds. However, the general trend—the slow killing strain survives at higher rates in small populations than in large populations— holds. Further, p = 1.0 is a very low threshold; it represents an average overlap of radii between two cells in contact of less than 0.4% of a cell radius. Thus, growth stops in large populations before the environment is densely packed, which prevents continued competition and either strain from overtaking the space. Thus, slow killing cells survive as they stop contacting fast killing cells.

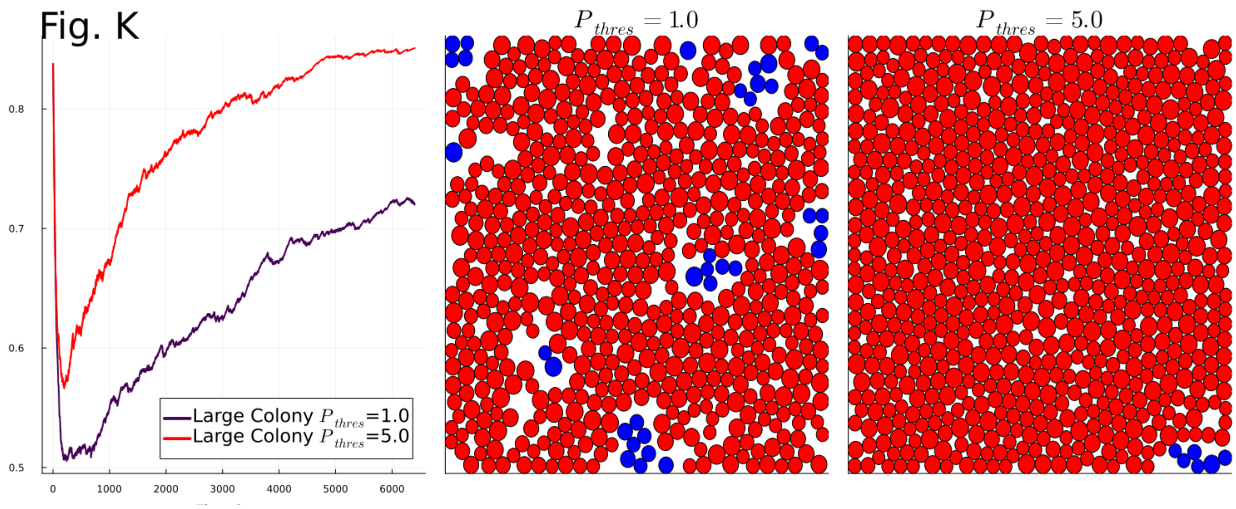

Fig. K The left panel plots the packing fraction against time.We see packing fraction drops rapidly and then slowly rises over time. However, at the end of the simulation it is far below the jamming threshold for the low pressure threshold and above it for the higher threshold. This leads the low threshold simulations to have large gaps between cells of opposing strains while the high pressure simulations allow the fast killing strain to overtake more space.The middle and right panels show visualizations of our simulations with the low and high threshold pressures after 64 generations.

Again, we note that even in these extreme cases where growth completely stops at a low pressure threshold, we still see the same general trends as described in our manuscript.

We were inspired by your comments to further expand on our model choices to see the extent of their effect on our results. We have now run simulations: for hard and periodic boundary conditions, where growth is limited by carrying capacity and packing fraction, where the cells start with different radii, and where there is a relaxation period before the simulation begins (cells start with minimal overlap)).

Further, we simulated all possible combinations of these approaches (including the simulations where growth is limited by pressure on individual cells), which represents 60 total approaches. For each approach, we simulated four population sizes. For each population size, we simulated 20 different killing ratios. For each killing ratio we simulated 500 replicates for the smallest size, 500 for the next largest, 50 for the next, and 5 replicates for the largest. In total we ran over 5 million new simulations. Displaying all of these data is a challenge, but we produced a plot like Figure 2 in the main text for each of these different

approaches.We have split each of the growth conditions into their own figure as it appears that this is the most impactful difference.

Fig. L

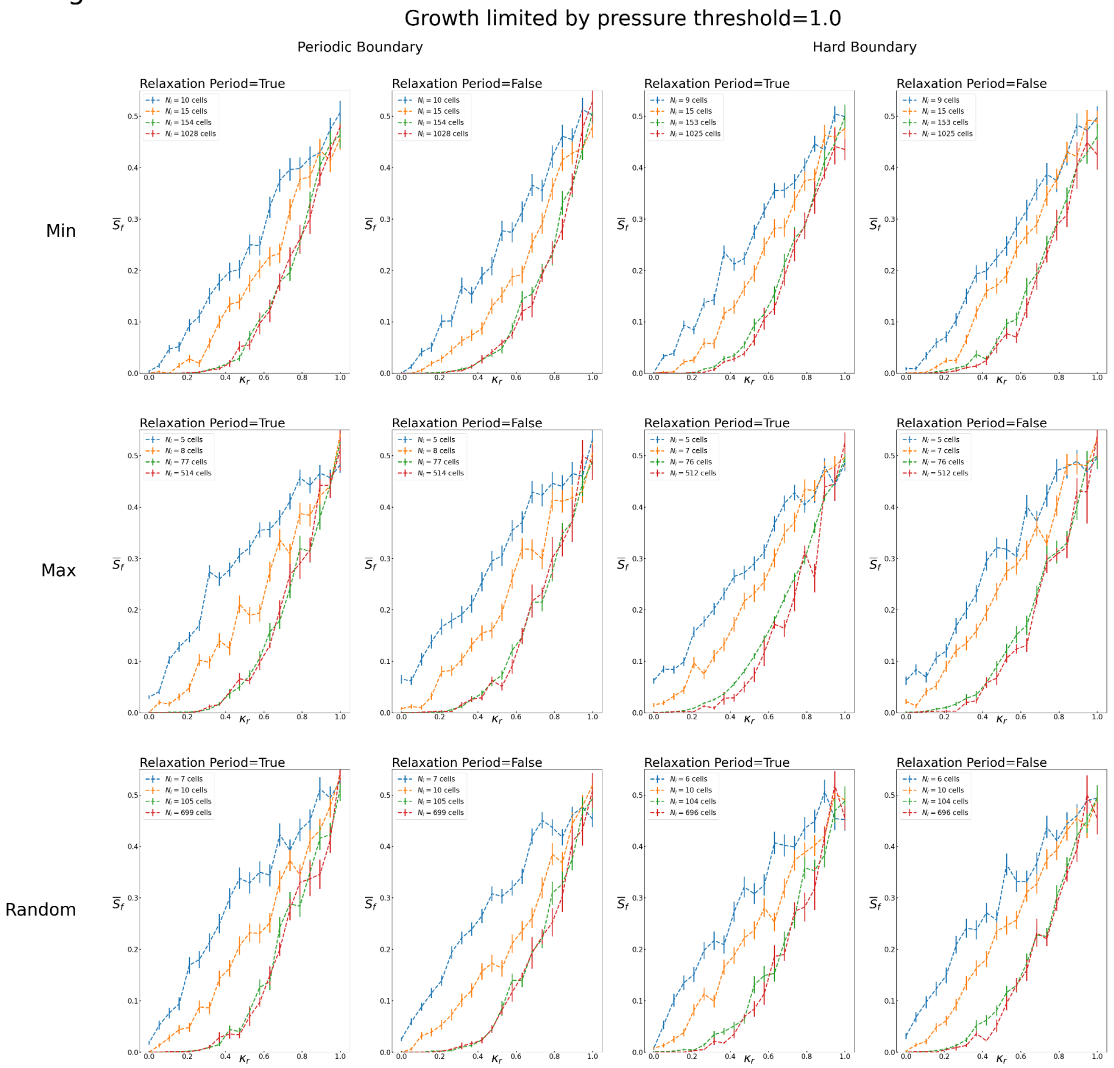

Fig L Above we show all parameter combinations of simulations where growth is limited by pressure on individual cells where the threshold is set to 1. We see that the effect described

in our manuscript is robust to how the simulation is performed; the small colonies always result in a significant increase in frequency.

Fig. M

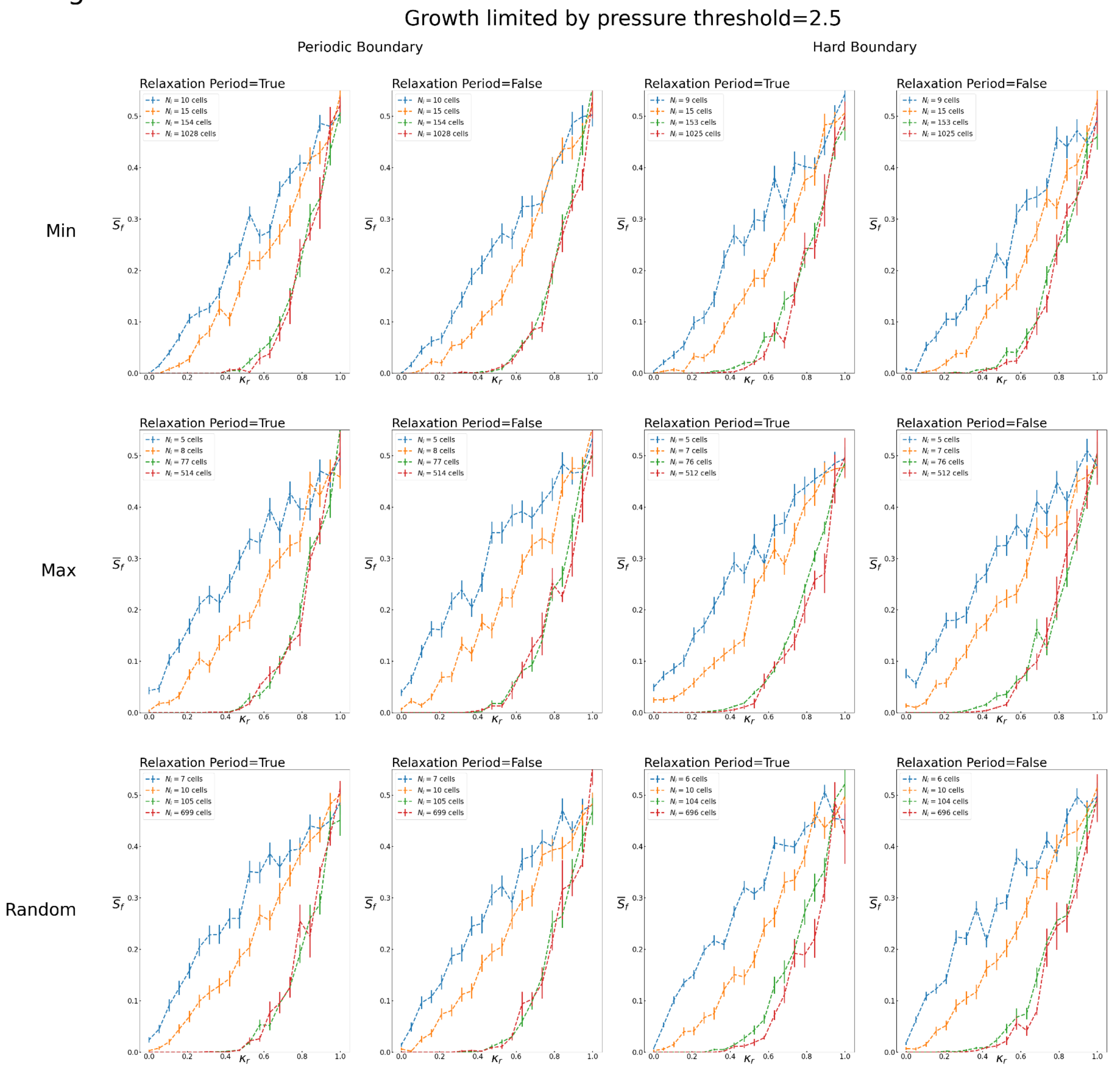

Fig M Above we show all parameter combinations of simulations where growth is limited by pressure on individual cells where the threshold is set to 2.5. We see that the effect

described in our manuscript is robust to how the simulation is performed; the small colonies always result in a significant increase in frequency.

Fig. N

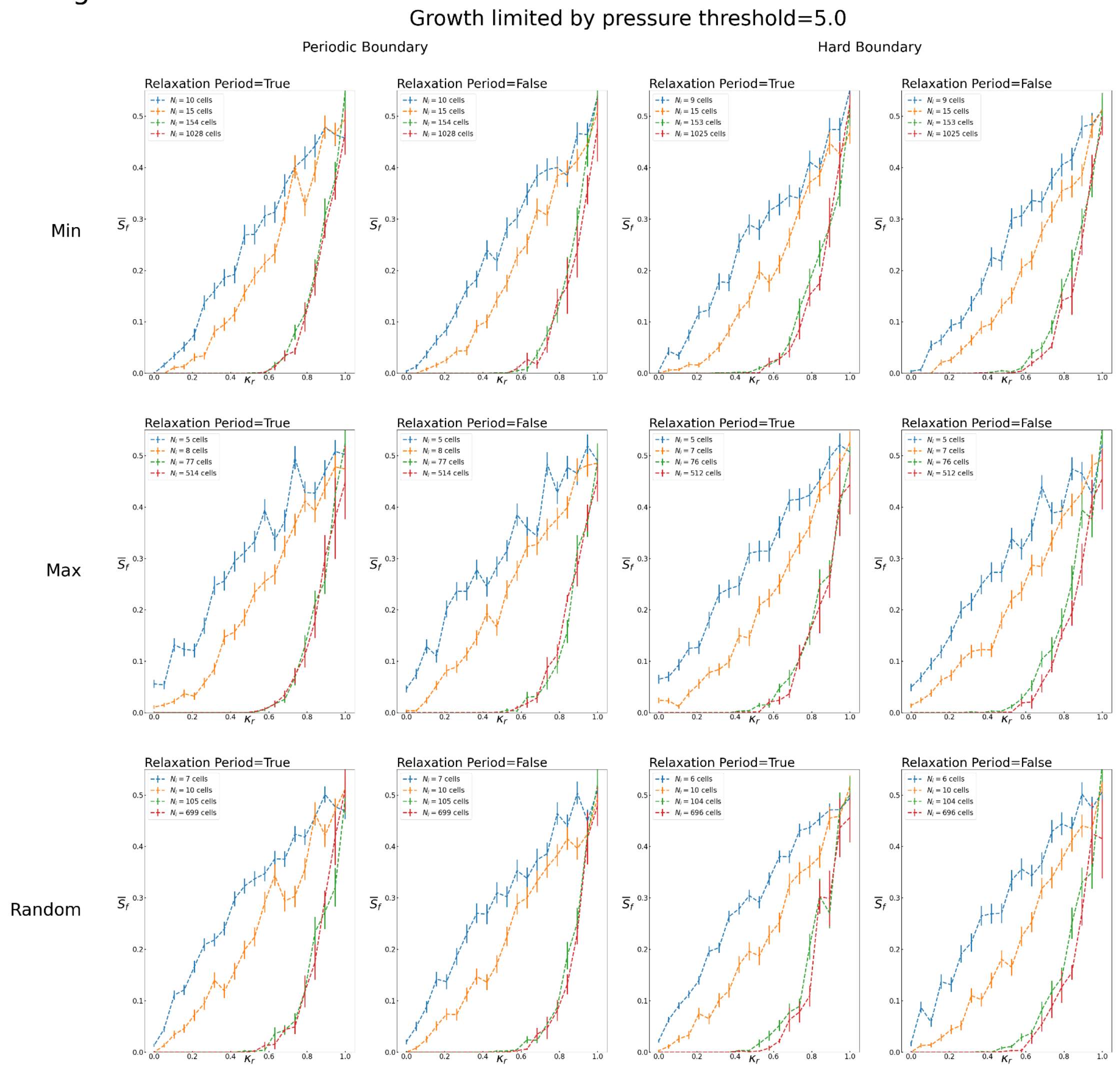

Fig N Above we show all parameter combinations of simulations where growth is limited by pressure on individual cells where the threshold is set to 5. We see that the effect described

in our manuscript is robust to how the simulation is performed; the small colonies always result in a significant increase in frequency.

Fig. O

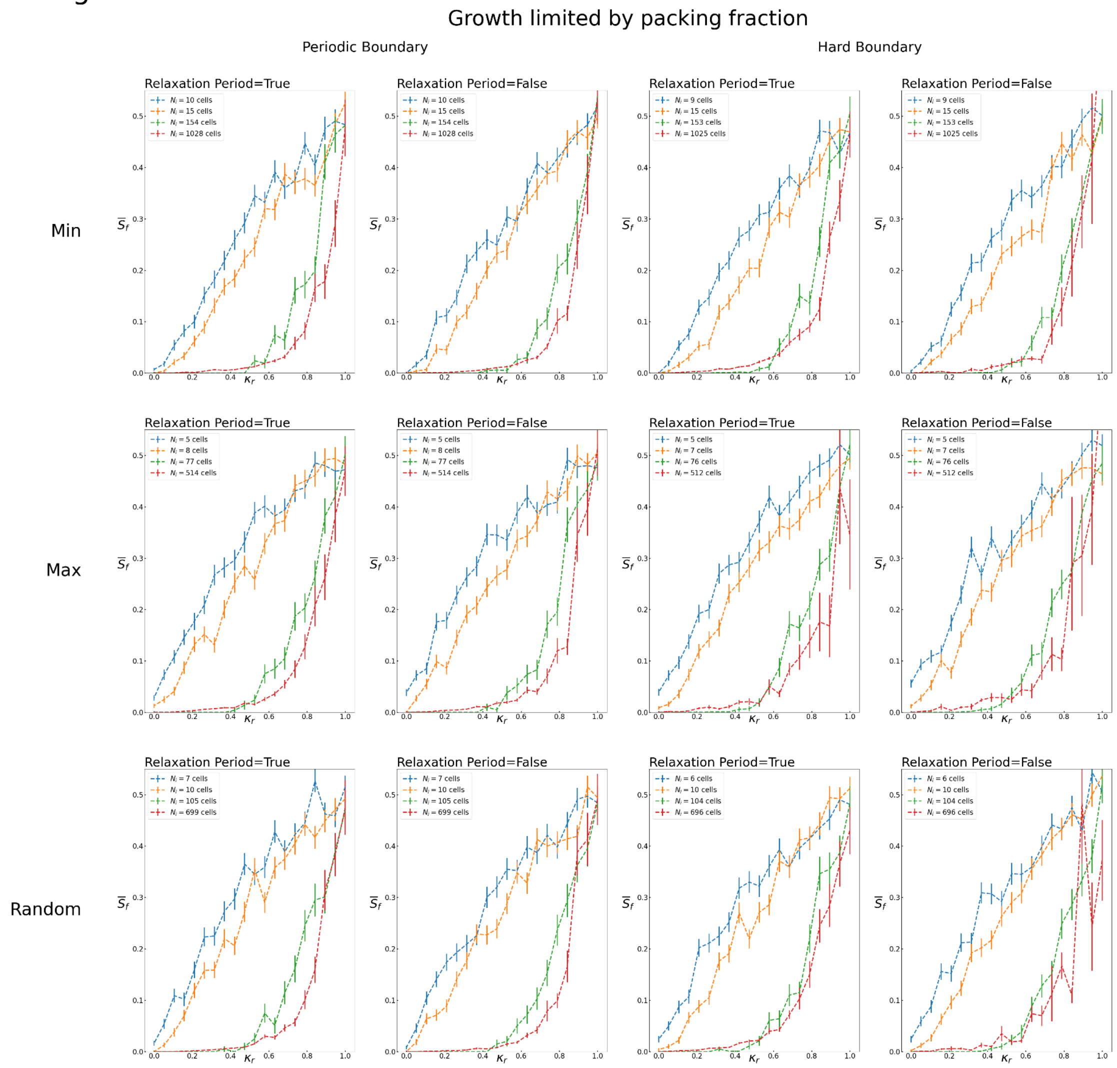

Fig O Above we show all parameter combinations of simulations where growth is limited by the packing fraction as described in our original manuscript. We see that the effect is robust

to how the simulation is performed; the small colonies always result in a significant increase in frequency.

Fig. P

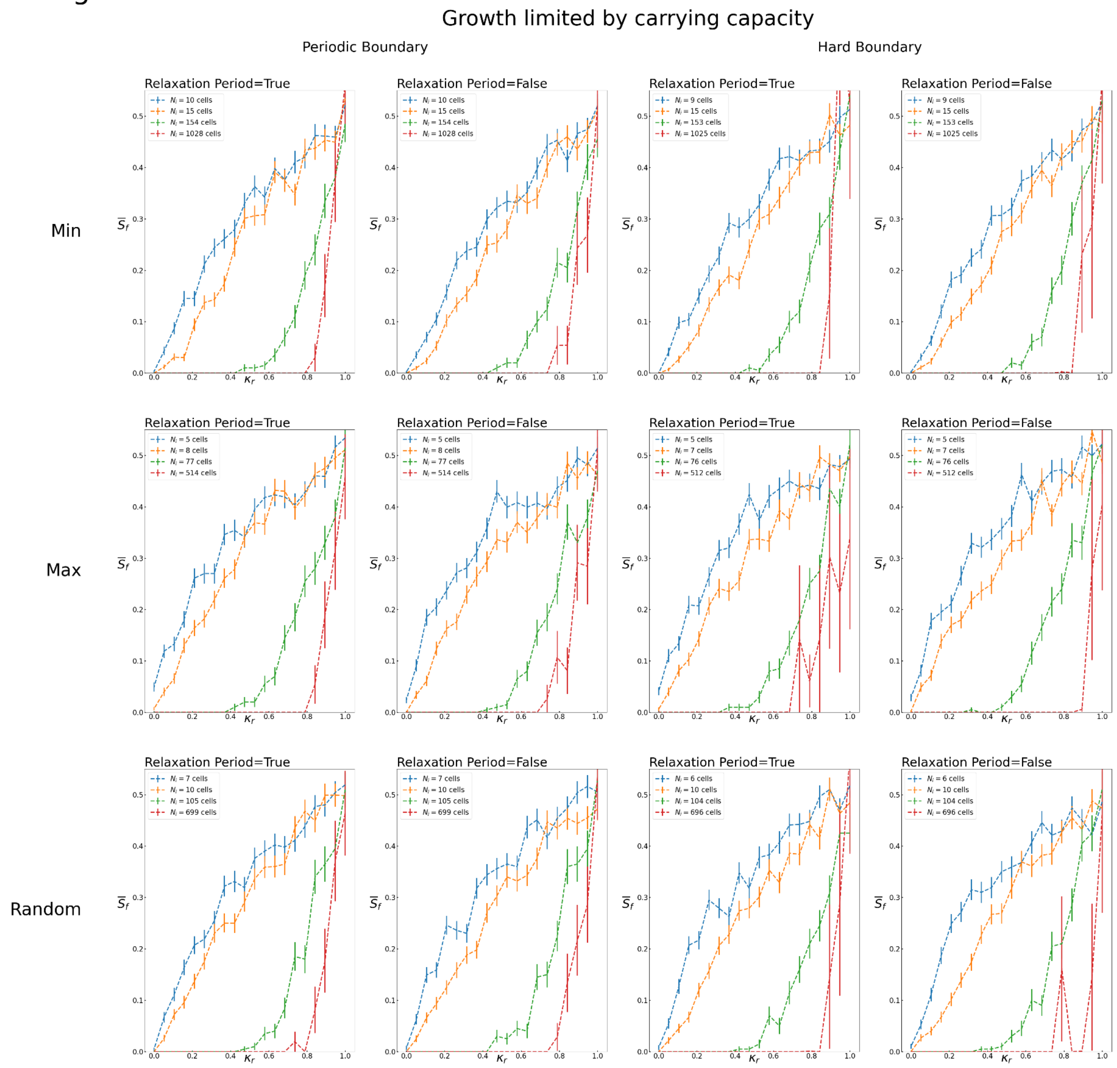

Fig P Above we show all parameter combinations of simulations where growth is not limited but rather a maximum number of cells for a given space is set. We see that the

effect is robust to how the simulation is performed; the small colonies always result in a significant increase in frequency.

We will go into detail about some of these results as we answer further comments below. However, we see that the smallest population always outperforms the larger populations. And while the exact details vary, it can be seen at a glance that the trends we report in the main text—enhanced survival in small populations and a ~linear S_f vs. k_r trend—are robust for the vast majority of the conditions (we will address the approaches that stand out below).

Thank you for suggesting investigating the effect of pressure on growth and how it may impact our results. We found that the phenomena we report are present for all modeling choices; further, there is almost no change in the results unless growth is heavily impeded by pressure then we recapture the same dynamics as before. Further, your comment led us to exploring all parameter choices in our model, and we believe it has improved the paper greatly as a result.

— Related to the previous question, what exactly is the stress at which growth is stopped at, and how does this compare to experimental results? All the model parameters are given in dimension-free values so comparisons to reality are hard to make.

Response: Thank you for this question. Given that the competitive dynamics occur at packing fractions below the jamming transition (as demonstrated in response to the previous question), measurements of stress are not relevant for the outcomes we focus on. However, this is an interesting question, so we will go into more detail below.

While we agree that comparing with experiments would be interesting, we want to emphasize that this is a difficult process. Recent papers (Gniewek, et al., Physical Review Letters 2019; Hartmann, et al., Nature Physics 2019) went to great extents to develop simulations and then experimentally validate many aspects of them. But even in those cases, they are only validated for a single organism and context. In particular, no one has experimentally validated simulations of *Vibrio cholerae* in spatially constrained environments. Simulations of budding yeast have been validated with deformable boundaries, and simulations of *Vibrio cholerae* have been validated in unconfined spaces. However, these approaches are substantially different, and thus even combinations of these approaches would need to be validated anew. Thus, comparing our stress values to

experiments would be inappropriate; regardless of if they agree, the interpretation would be unclear (e.g., the means could match though the distributions are different).

In a different vein, we have previously demonstrated that the outcomes of contact killing competitions can be accurately simulated with individual based models like the one we use here (as well as a wide range of other approaches, see McNally, et al., Nature Communications, 2017); this observation was validated and made much more rigorous by a subsequent paper (Lavrentovich and Nelson, Physical Review E, 2019). As our interest here is in the outcome of said competitions, we are not performing simulations that are experimentally validated for other quantities, such as stresses.

However, we agree that it is definitely worth exploring the stresses within our simulations, especially to ensure that they are not non-physically large. As described above, the simulations we report stop growth when the model reaches the maximum packing fraction. We first measured the average pressure on cells in our simulations at the end of 64 generations for the small and large colony sizes and the parameter choices in the original manuscript (Fig Q).

Fig. Q

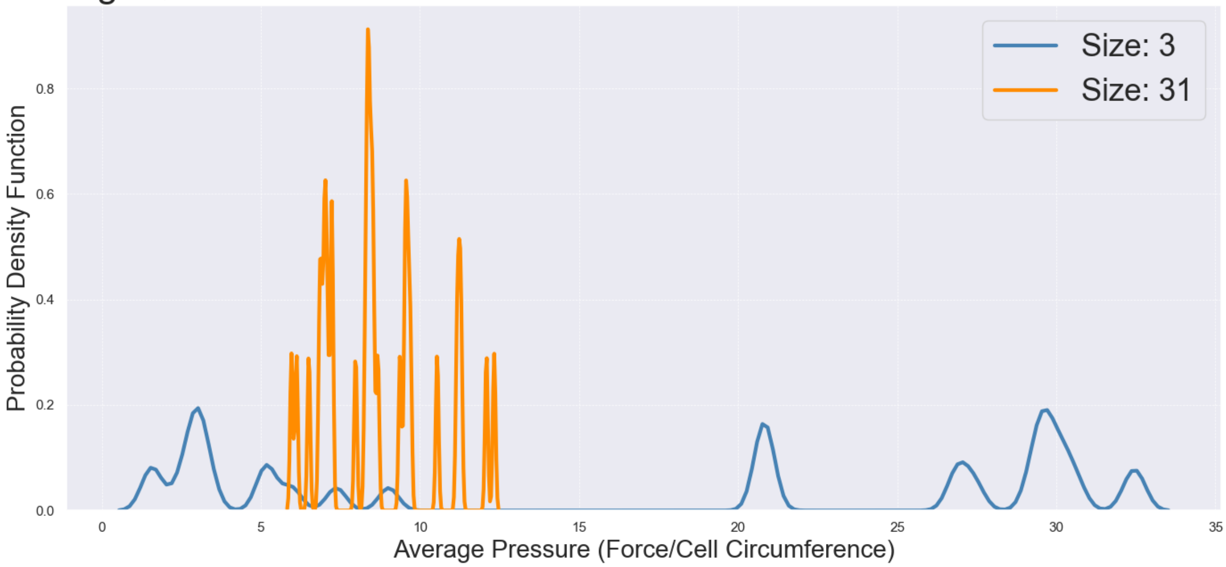

Fig Q Here we show the probability density function of the average pressure on the remaining cells after 64 generations. Size 3 and 31 corresponds to 9 and 1025 cell simulations respectively. Each trend is made of 30 data points, and the parameters are as

follows: hard boundary conditions, growth is stopped upon global packing fraction exceeding the threshold described in the our original manuscript, no relaxation period, cells start with the minimum radius. The small colony size explores a large range of values than the larger as the cells can jam in non-optimal configurations with the wall resulting in large pressures.

The larger pressures are coming from simulations with hard boundaries in small spaces. To add more context to these numbers, a pressure=1 is equivalent to having a net overlap with other cells of 0.013 as a proportion of cell radius (i.e., a total of 1.3% overlap). For pressure = 5, the equivalent is a net overlap of 0.063 proportion of radius (i.e., a total of 6.3% overlap). As the average cell will have four neighbors, this equates to 0.4% to 1.5% radial overlap per neighbor.

— A single time step dt=0.01 is used in the simulations. But given the nature in which the contact killing is incorporated in the model, it will be important to show that the results are converged and insensitive to changes in the value of the time step used. This does not appear to have been done.

Response: You are correct, this is an important point to address. An incorrect time step choice could introduce artificial stochasticity into the results. The only source of stochastic rates in our simulations are killing events.

Fig. R

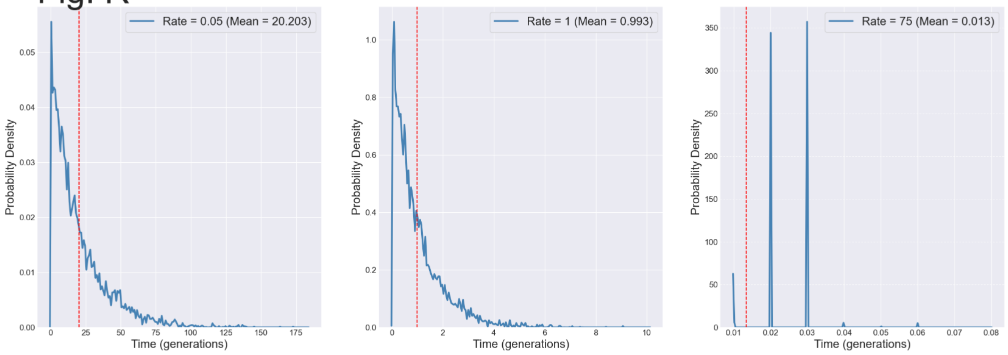

Fig R We show the probability density function for the time for a killing event to occur given killing rates of 0.05 (left panel), 1 (middle panel), and 75 (right panel) events/generation; each panel is the result of 10,000 data points. The left and middle panel are the largest and smallest killing rates we test in our paper. To compare an inappropriate choice of killing rate and time step, we also show the average time to kill for a killing rate of 75 events/generation (right panel).

We see in Fig R that the time step used in our paper produces a smooth pdf curve while the rate=75 curve is jagged and a clear demonstration of a poorly chosen rate/dt.

— Also, it would be useful to show that the results are insensitive to the choice of the shape of the square boundary e.g. use a circle, since a square introduces sharp edges.

Response: Thank you for this suggestion. We agree that testing the effect of sharp edges and corners in the boundary is an important control. Rather than code a circular boundary, we simulated periodic boundary conditions that lack edges of any kind. Above we show that the results are insensitive to periodic boundary conditions; that plot is reproduced here:

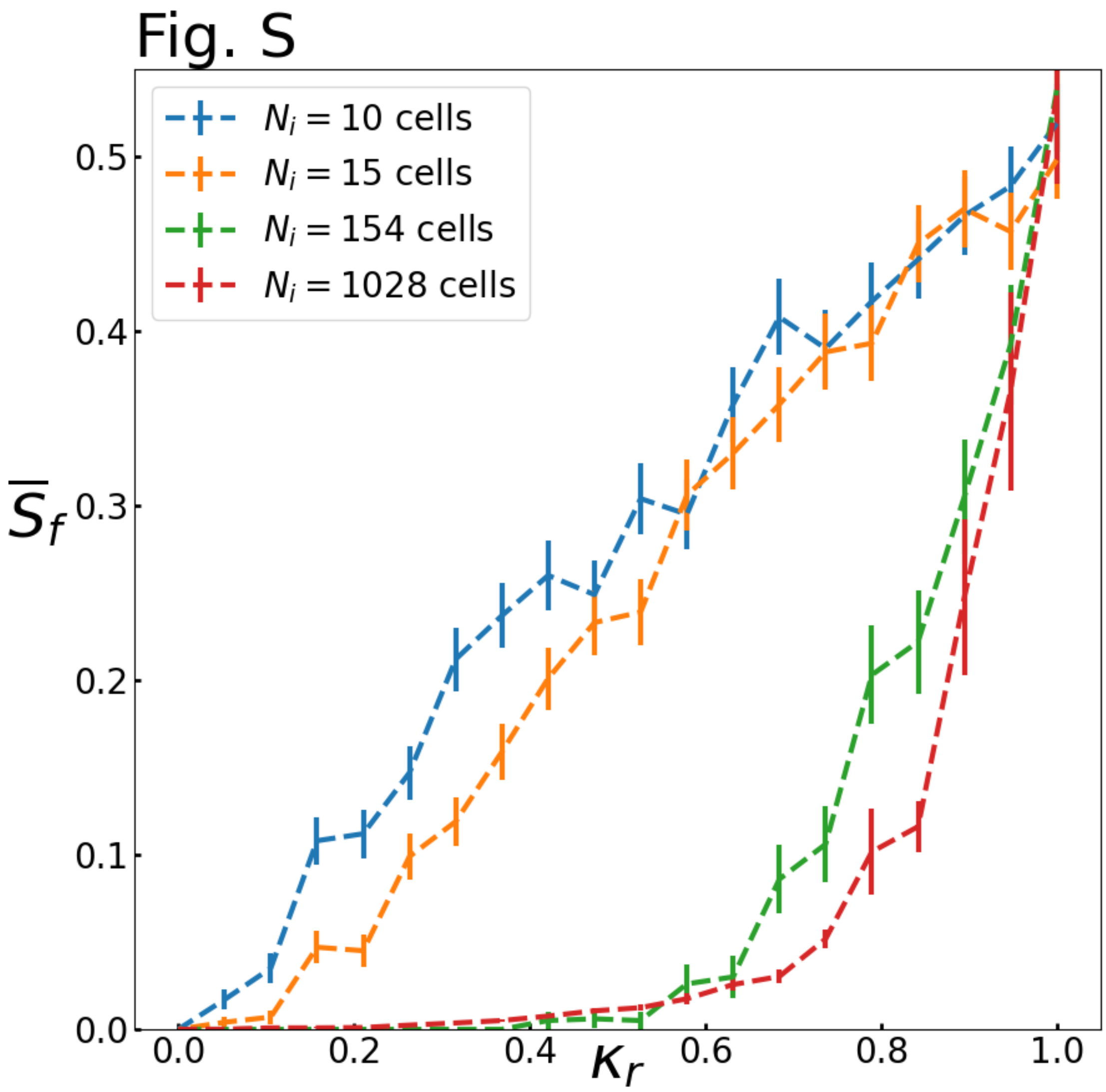

Fig S The simulations shown in this plot have all the same parameters as the original manuscript except for the boundaries, which are now periodic. We find the same trend as reported where the small colonies yield a larger frequency of the slow killing strain.

Periodic boundary conditions lack the sharp edges and straight lines of the square boundary, and yet show similar phenomena, suggesting that our results are not

substantially affected by the system boundary conditions. We now make this point in the manuscript.

— Related to this point: it would be useful to actually show images from the simulations of the spatial distributions of the cells, instead of just abundances. How do these compare to the spatial distribution and sizes of the different aggregates in the experiments? A more rigorous comparison to the experiments would be useful.

Response: We agree, showing images of our simulations is a very helpful idea. Further, it is important to show that our simulations capture key aspects of the experiments, particularly the spatial effects. It can be seen in our experimental images that the cells phase separate into aggregates—this is a key difference between spatial and non spatial models. We have added images from our simulations to show this effect has been captured in them, much like in the experiments.

Fig. G

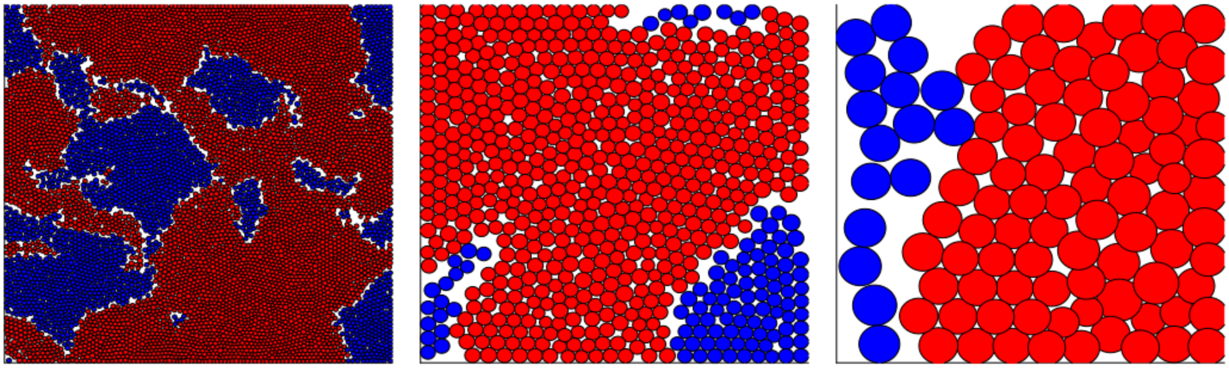

Fig. G Each panel shows a visualization of our simulations when killing rates are similar after 40 generations. The panels show 10,000, 1000, and 100 cells respectively.

However, while the simulations and experiments are visually similar, our goal for the experiments was simply to test if we can observe the slow killing strain surviving more in small spaces than in large spaces. To make direct quantitative comparisons, we would have to modify the simulation to better replicate *Vibrio cholerae* (see, for example, this paper: Hartmann, Raimo, Praveen K. Singh, Philip Pearce, Rachel Mok, Boya Song, Francisco Díaz-Pascual, Jörn Dunkel, and Knut Drescher. "Emergence of three-dimensional order and structure in growing biofilms." Nature physics 15, no. 3 (2019): 251-256.).

— The authors define kr = kf/ks, which should be greater than one, but this appears to have been flipped in the actual presentation of the results.

Response: Thank you for pointing out this typo; it has been corrected.

— In Fig 3, why are the values of the horizontal axis different between A-B and C-D?

Response: This is due to the fact that frequency changes in these populations in discrete steps. When N is large, these steps are very small (and can appear almost continuous). However, when N is small, these discrete steps are larger (a single cell could represent 16% of the population).

— The authors discuss Fig 2 and say "there is a critical value for the killing ratio". Is there? Or is it a gradual increase in the fraction? The authors would do better to be more precise in their language here and in other places.

Response: Thank you for your comment. In light of this and the expansion of our model above which showed that this effect varies under different model choices, we have revised our language.

— The supposed shift shown in Fig 3 is a very weak effect. If anything, it just looks like the boundary becomes more diffuse in the case of the smaller system, as one might expect given the increased importance of fluctuations. Again, it would be useful for the authors to discuss this more carefully and be more precise in their language.

Response: Thank you for your comment; we agree, this topic could use more clarification, and we have modified our language to reflect this.

— In the experiments, have the authors verified that the growth rates of the different strains are identical? Otherwise they are changing multiple variables at one time.

Response: We measured the growth rate of each strain using a BioTek Synergy H1 plate reader. We found that the differences in growth rate were not significantly different. The growth rate of the strain shown in blue is 34.92 +/- 0.96 minutes per generation (n = 24) and the growth rate of strain shown in yellow is 35.95 +/- 0.87 minutes per generation (n = 24).

— In Fig 7, the authors write 22.2% and 30.3%. What do these numbers mean exactly? What is the uncertainty? Do they really have precision to sub percent resolution?

Response: These are the mean frequencies of the strain shown in red which was calculated by finding the number of pixels that were red divided by the total number of pixels. The number of pixels analyzed in each image was 262,144; each pixel is from 0.59 to 1.65 microns wide, and thus can contain approximately ~1 Bacteria. Thus, we likely are imaging hundreds of thousands of bacteria.

— In the text discussing Fig 8 the authors write values like -0.048 +/- 0.023. How do they have uncertainty to two significant figures? That does not appear to be meaningful.

Response: Similar to the above response, we analyze 262,144 pixels for each replicate (representing hundreds of thousands of bacteria).

— What do they error bars in Fig 8 represent?

Response: The error bars represent the standard error; thank you for pointing this omission out. A description has been added clarifying this.

— The experiments, while promising, don't show any suitable negative controls. Eg. what happens when you just have one strain growing in each geometry (TEM grid versus without).

Response: Thank you for raising this question. We agree that to properly interpret the experiments it is crucial to show that the TEM grids are not negatively impacting the growth of one strain relative to the other. In particular, as we see the slow killing strain performs better in the small TEM grids than it does on open agar, it is important to show that the TEM grids do not inhibit the growth of the fast killing strain.

Unfortunately, monitoring growth in this fashion across time scales is technically difficult. For our imaging set-up, fluorescent proteins start out bright, but then dilute after many rounds of replication; as the rate of replication slows, FPs accumulate in the cells that are present, making them bright and easy to image again. Thus, while we observe no difference in the growth dynamics at early and late times, the missing information in the middle makes this an imperfect control.

Thus, we instead focus on a different control–larger TEM grid spaces. We performed a new series of experiments in TEM grids with square openings of length 60 microns, a hundred fold increase in area. Thus, if the TEM grid inhibits the growth of the fast killing strain, then we would expect to see the relative abundance of the slow killing strain end closer to that of the small grids than that of open agar.

Fig. T

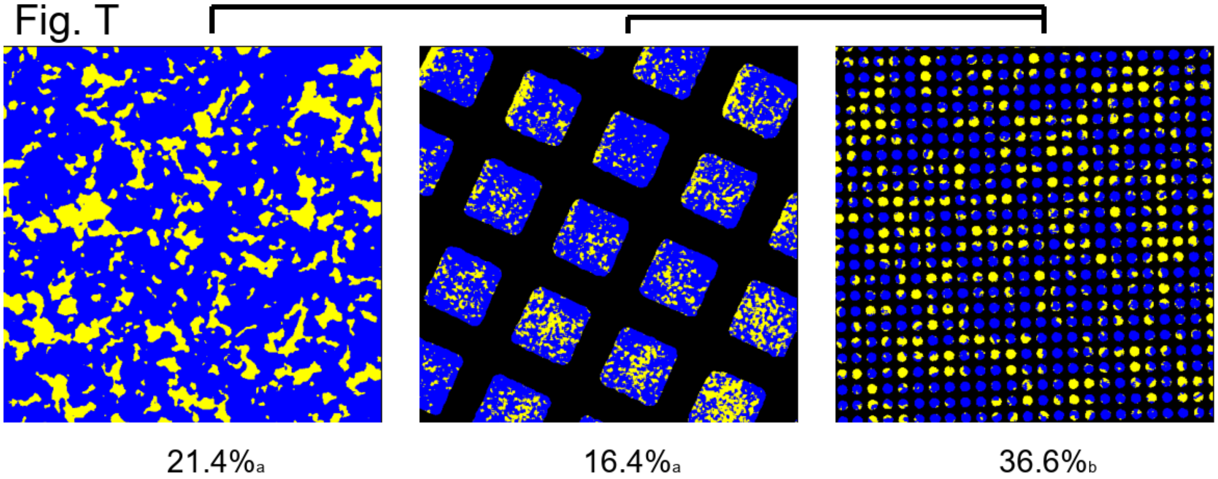

Fig T We show two strains of *Vibrio cholerae* 6 hours after inoculation with an initially equal proportion of “blue” and “yellow” competitors on open agar (left panel), in grids with large spaces (~60 by ~60 microns, middle panel), and grids with small spaces (~7 by ~7 microns, right panel). The dimensions of each image are the same (~300 microns), and the fast killing and slow killing strains are colored blue and yellow, respectively. The slow killing blue strain ends with a higher frequency in the small grid condition than the two larger spaces. Subscripts show significance groups and connected images show significant differences ($p<0.05$). Each panel is the result of 5 data points.

Instead, we find that the open agar and large grid outcomes are within standard error bars of each other, and their difference is not statistically significant. Further, even if it were a real effect it would suggest that the TEM grids inhibit the slow killing strain, which would make the substantial increase in the slow killing strain in the small grids an even stronger effect.

— The authors mention biofilms in the abstract and throughout the paper. But it is not clear how the matrix produced by biofilms would influence the results presented here, since presumably that would hinder the contact-mediated killing. This feature is not explicitly

considered in the model nor is it clear that the colonies studies in the experiments are actually secreting a matrix (staining would help verify this). It is not clear if the connection to biofilms is even necessary to be made here.

Response: Defining exactly what is and is not a biofilm is notoriously difficult. The strain of Vibrio cholerae, and its derivatives we use here, do secrete EPS (Hammer & Bassler 2003 mBio https://doi.org/10.1046/j.1365-2958.2003.03688.x) and is a model for studying biofilm formation and architecture. However, we do not simulate the extracellular matrix, and so for the sake of clarity, we agree that the term “colony” would be appropriate. We have replaced all instances of the word biofilm with colony throughout the manuscript.

— Minor point: What exactly is shown in Figure 1A? Labels would be useful here. How was this sample prepared? How was it imaged? What color is what? What are the big black circles? Why is the purple diffuse and the green in spots? This image is completely unclear.

Response: Figure 1 A is adapted from (Probandt D, Eickhorst T, Ellrott A, Amann R, Knittel K. Microbial life on a sand grain: from bulk sediment to single grains. The ISME Journal. 2018;12(2):623–633. doi:10.1038/ismej.2017.197.) and shows a well imaged grain of sand where bacteria fluoresce green. Upon reinspection, we agree that this could use more discussion and context. Further, we are removing figure 1 B for clarity and simplicity

Reviewer #3: This is an interesting manuscript that analyzed the effect of population size and random sampling in competitions between antagonist bacteria. The main question, whether small population sizes may alter the outcome of antagonism between such strains, is an important one, given that many microbial populations (e.g., in soil, in the intestine, in skin pores, etc.) are small and spatially separated from others.

I have a few major points that I would like to see addressed before recommending publication:

Response: Thank you for your kind words and insightful comments. We believe that addressing your comments improved the manuscript.

On the experimental side, I commend the authors for pursuing experimental verification of

the results. I am worried, however, that the physiological condition of strains in the two setups may be quite different, possibly leading to differences in the antagonistic interaction dynamics. I would suggest, if possible, to perform also the large-population competitions in TEM grids of different sizes, rather than on agar plates. This would ensure that the bacteria in the two treatments are in comparable states.

Response: Thank you for this suggestion. It is important to show that the TEM grids are not introducing any physiological effects that may impact the strains. We agree that conducting experiments in TEM grids with larger spaces is a great way to deal with this potential issue.

Fig. T

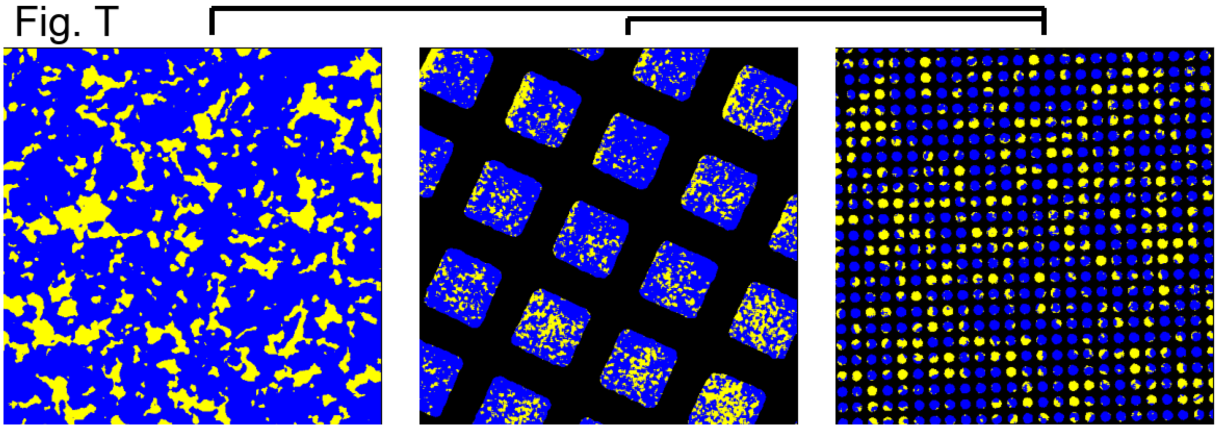

Fig T We show two strains of *Vibrio cholerae* 6 hours after inoculation with an initially equal proportion of “blue” and “yellow” competitors on open agar (left panel), in grids with large spaces (~60 by ~60 microns, middle panel), and grids with small spaces (~7 by ~7 microns, right panel). The dimensions of each image are the same (~300 microns), and the fast killing and slow killing strains are colored blue and yellow, respectively. The slow killing blue strain ends with a higher frequency in the small grid condition than the two larger spaces. Subscripts show significance groups and connected images show significant differences ($p<0.05$). Each panel is the result of 5 data points.

The open agar and large grid outcomes are within standard error bars of each other, and their difference is not statistically significant. Further, even if it were a real effect it would suggest that the TEM grids inhibit the slow killing strain, which would make the substantial increase in the slow killing strain in the small grids an even stronger effect.

On the theoretical side, I wonder how robust the linear trend observed in Fig. 2 for N_max = 9 is to changes in the parameters, most importantly the total simulation time. Intuition would suggest that these curves, especially those for large populations, are not representative of the infinite-time limit, in which due to competitive exclusion we would expect either one or the other strains to completely colonize the landscape, but stochastic sampling of the initial condition may allow \bar S_f to be different from zero. Is the linear trend observed for N_max = 9 simply due to stopping the simulations early, or is it representative of the infinite-time limit? How do these curves look like in the absence of sampling, i.e. with a fixed S_i = 0.5?

Response: You are right in suspecting that the large population trend is not the infinite time limit representation. Below are examples of the frequency over time, and we think they show that the length of the simulations was appropriate as the small spaces all go to fixation while the large spaces hit a stable frequency.

Fig. E

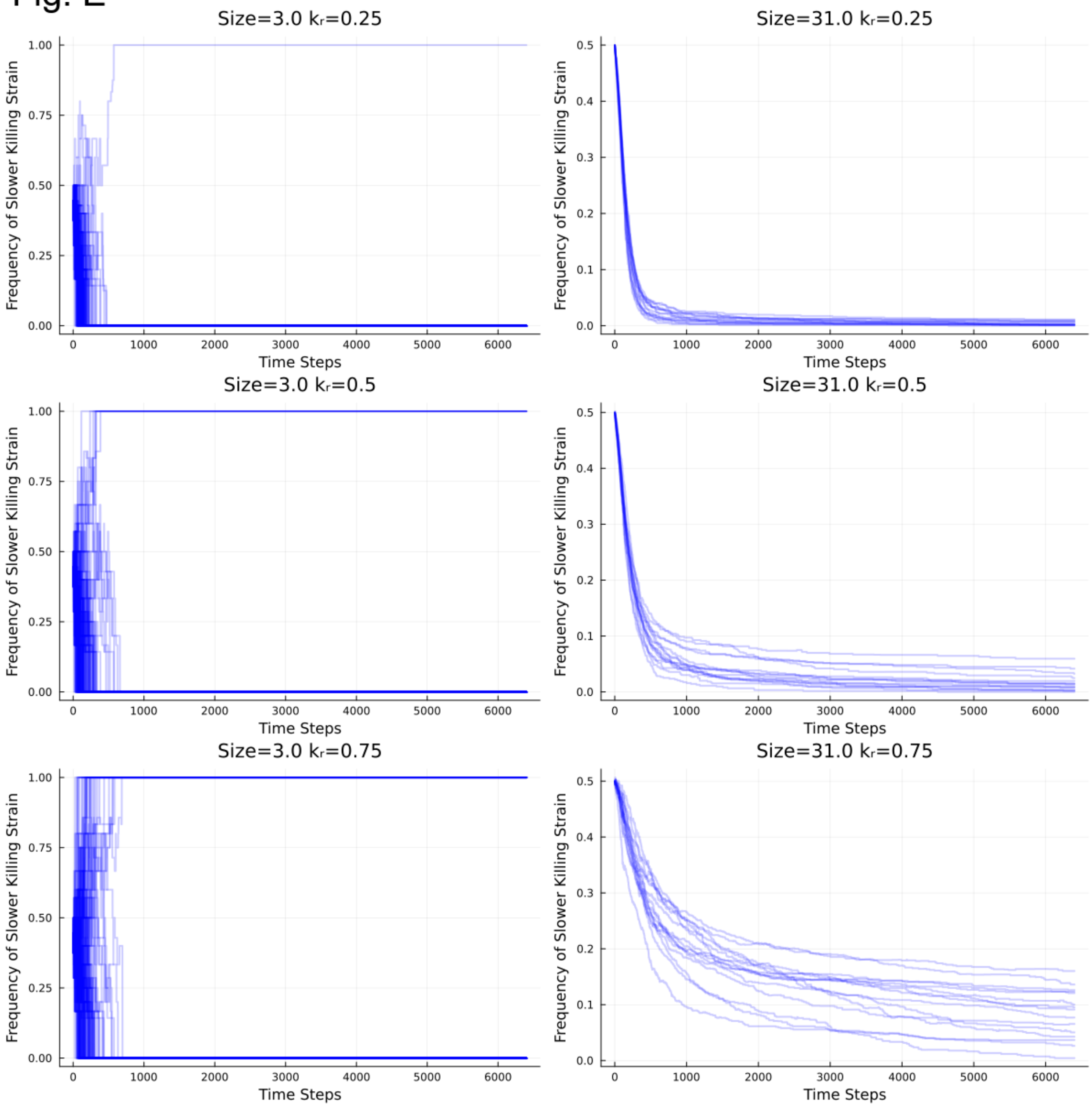

Fig. E The frequency of the slow killing strain is plotted versus time steps; every 100 time steps is a generation as this is the average time taken for one cell to divide. All panels show 15 trends. Size 3 and 31 correspond to 9 and 1025 cell simulations respectively.

We agree that fixing S_i may reveal whether we have correctly identified the cause of this effect and have investigated it.

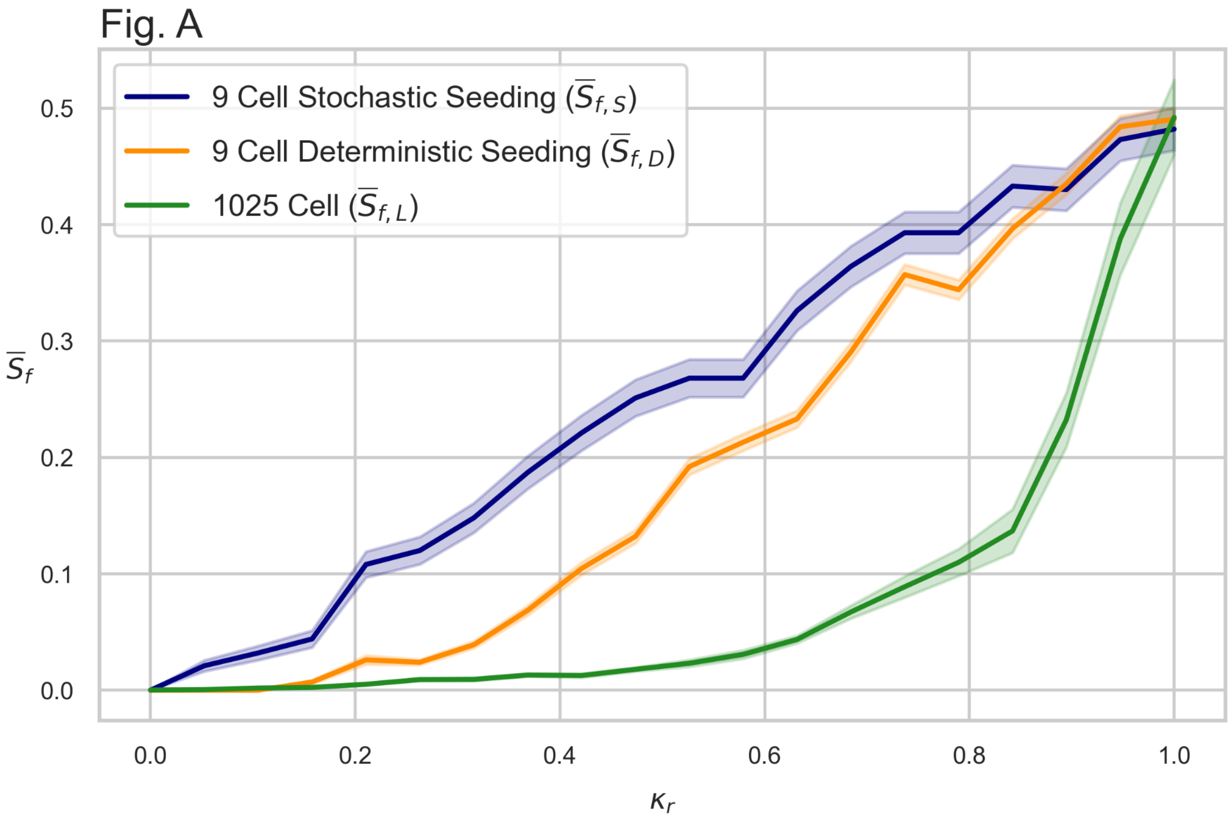

Fig. A. S_f is the final proportion of the slow killing strain, and k_r is the killing rate ratio of the slow killing strain to the fast killing strain. Stochastic seeding means that the initial abundance of the slow killing strain was randomly selected from a binomial distribution with equal chance of either strain and the total cell count = 9. Deterministic seeding with an equal proportion is impossible for 9 total cells, so we averaged the trends where the initial abundance of the slow killing strain is held at 4 and 5 cells out of 9. There are 20 data points in this trend line; each point is the result of 1000 simulations. These two lines describe simulated “small colony” sizes. The 1025 cell simulations are also stochastically seeded; the initial proportion is expected to be 0.5 +- 0.016. There are 20 data points in this trend line, and each is the average of 50 simulations. This line describes a simulated “large colony”. The error bars represent standard error.

From Fig A we can quantify direction how much of the finite size effects are due to seeding (see Fig B below)

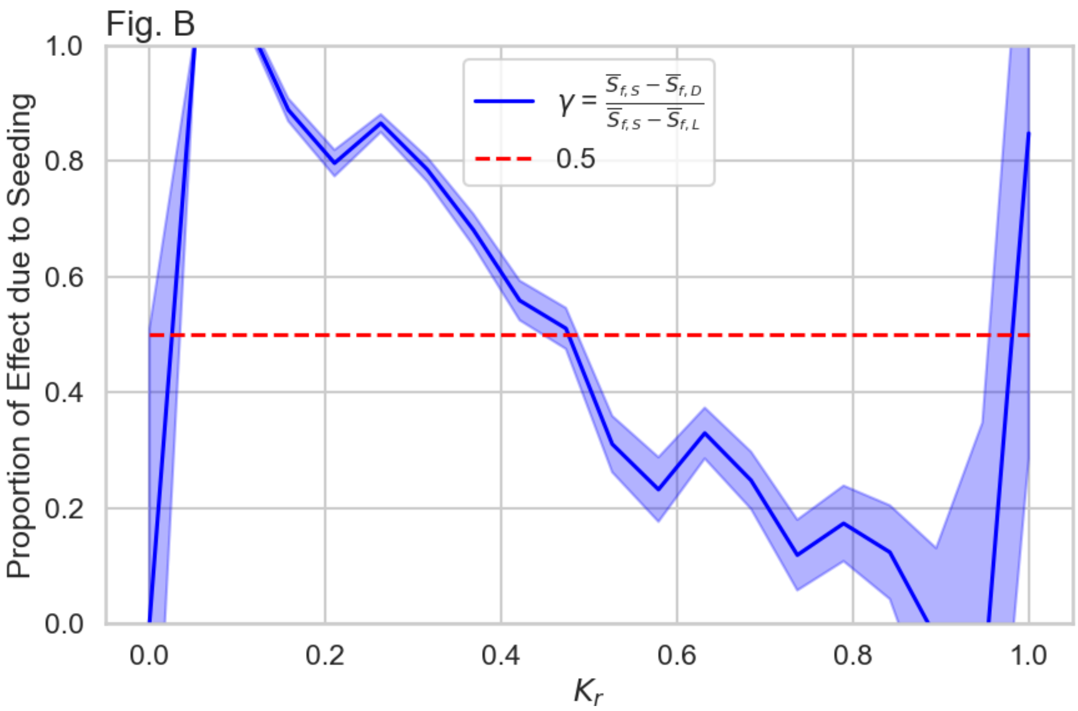

Fig. B K_r is the same killing ratio shown in the previous figure (Fig. A), and ɣ is the difference between the stochastic and deterministic small colony seeding trends divided by the difference between the stochastic, small colony and the large colony trends. This quantifies how much of the "total finite size effect," i.e., the difference between large and small simulations, is a result of stochastic seeding.

We find that especially at low Kr the seeding effects are the most important effect leading to the survival of the slow killing strain. Other factors contributing to the overall finite size effects include: initial position of cells, stochastic killing rates, and the inability of the fast killing strain to use its advantage to reach isolated populations. However, we have further shown here that seeding plays a major factor in almost all conditions. See Figure C.

Fig. C

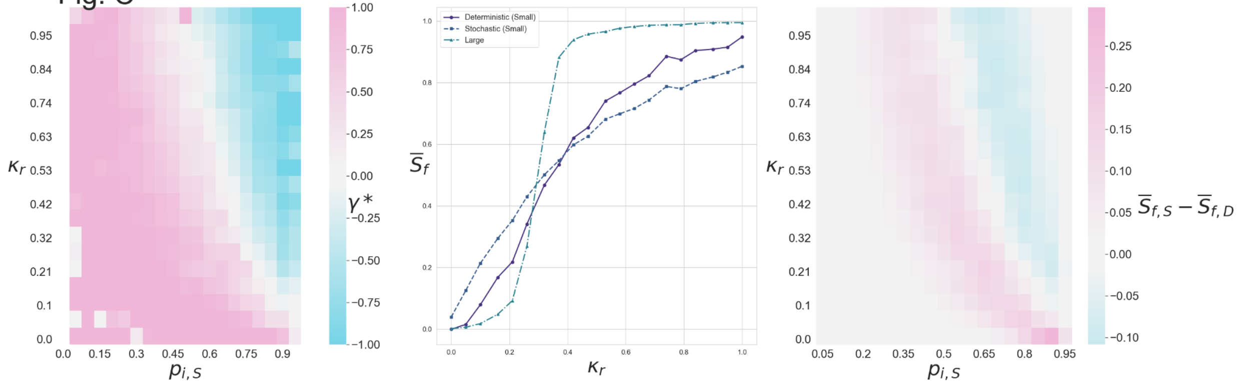

Fig. C. In the heatmaps above (Fig. C) , P_i,s is the initial proportion of the slow killing strain in the planktonic suspension, and K_r is the ratio of the killing rate of the slow killing strain to the killing rate of the fast killing strain. S_f in the middle panel is the final proportion of the slow killing strain after 64 generations averaged over 1000 simulations for the small colony sizes and 30 for the large. In the left panel, we plot γ^* on the z axis. It is calculated by taking the ratio of the final fraction of the slow killing strain in stochastic seeding conditions minus the final fraction of the slow killing strain in deterministic seeding conditions to the larger of:

3. the final fraction of the slow killing strain in stochastic seeding conditions in small populations and the final fraction of the slow killing strain in large populations
4. The final fraction of the slow killing strain in deterministic seeding conditions and the final fraction of the slow killing strain in large populations.

Mathematically, this is calculated as:

$$\gamma^* = s\_f,s - s\_f,d \ / \ MAX(|s\_f,s - s\_f,L|,|s\_f,d - s\_f,L|)$$

This is similar to gamma introduced above. The sole difference is that the denominator is now the absolute value of the largest difference of either option 1 or 2. We made this change as sometimes the stochastic seeding does not aid the slow killing strain but actually impedes it (See Fig C middle panel). While our original manuscript reported that finite size effects aid the fast killing strain when at a numeric disadvantage, here we can see that a major factor behind that observation was stochastic seeding effects.

We see that seeding has a major impact across these conditions, and in fact, it is greater than 0.5 or less than -0.5 in 90% of conditions. However, this proportion does not reflect how large the effect is. Therefore, we show the difference between stochastic and deterministic seeding in small populations in the right panel of Fig. C. We see that as before, the importance of seeding increases as k_r decreases and can lead to a total frequency increase of 30%. These graphs will go in the supplementary material.

The authors have singled out the effect of sampling by performing simulations with fixed initial conditions, namely the relative abundance of the two strains, but I feel that the cause of the increased survival of the slow killers in small populations hasn't been completely identified. It could be simply due to stochastic fluctuations in small populations, but Fig. 7B makes me wonder what is the effect of space and in particular of the boundary conditions: small populations will have relatively more cells at the boundary, with fewer neighbors than in the bulk, compared to large populations. This might have a big effect in terms of prolonging the survival of the slow killer, especially if found at the boundary. To single out the effect of boundaries, one possibility would be to repeat the simulations with periodic boundary conditions.

Response: Thank you for this comment. We agree that this serves as a good comparison for our model. We have done these simulations and now show them in the supplementary material (and below) and found they did not significant;y impact our results. Please note the cell number is different simply because this was one of many new simulations we ran for our responses.

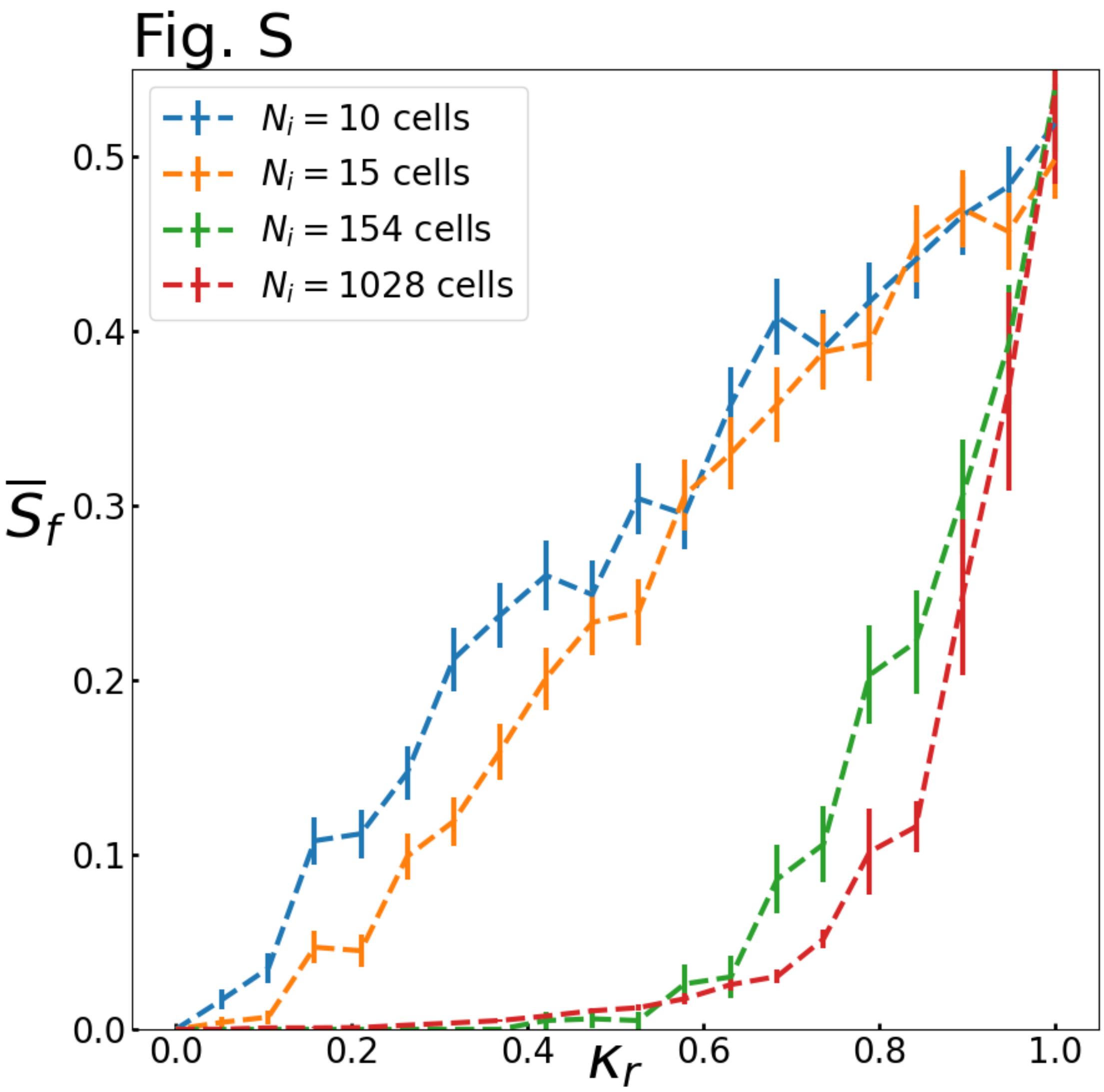

Fig S The simulations shown in this plot have all the same parameters as the original manuscript except for the boundaries, which are now periodic. We find the same trend as reported where the small colonies yield a larger frequency of the slow killing strain.

We have investigated many more model choices and found that the reported effects are robust (see SI for details).

It is unclear if the \bar S_f values plotted in Fig. 3 all come from simulations in which either one of the two populations has fixed (i.e., S_f = 1 or S_f = 0) or not. Does the statement 'there are more initial conditions that enable the survival of the slow killer in the small system than there are in the large system' hold when only data from simulations in which fixation occurred are used? In other words, do stochastic fluctuations (at fixed S_i) allow the slow killer to survive in a broader region of parameter space asymptotically in time, or do they allow it to survive for longer times? Fig. 6 suggests that the latter may be true. These same questions apply to Fig. 5

Response: Thank you for raising this question. We agree, it is crucial to understand the temporal dynamics. We find that small populations (N=9) always end with go to fixation and are very stochastic, while the large populations often end in a steady state with both strains and are highly deterministic.

Below we show plots of frequency over time for three killing ratios for small populations (N=9) and large populations (N=1000). These plots show that the small populations only end with one strain, while the large populations often end in a steady state with two strains. Thus, the enhanced survival in small populations is not due to slow killing cells surviving longer, but to the slow killing strain actually winning more often.

Fig. E

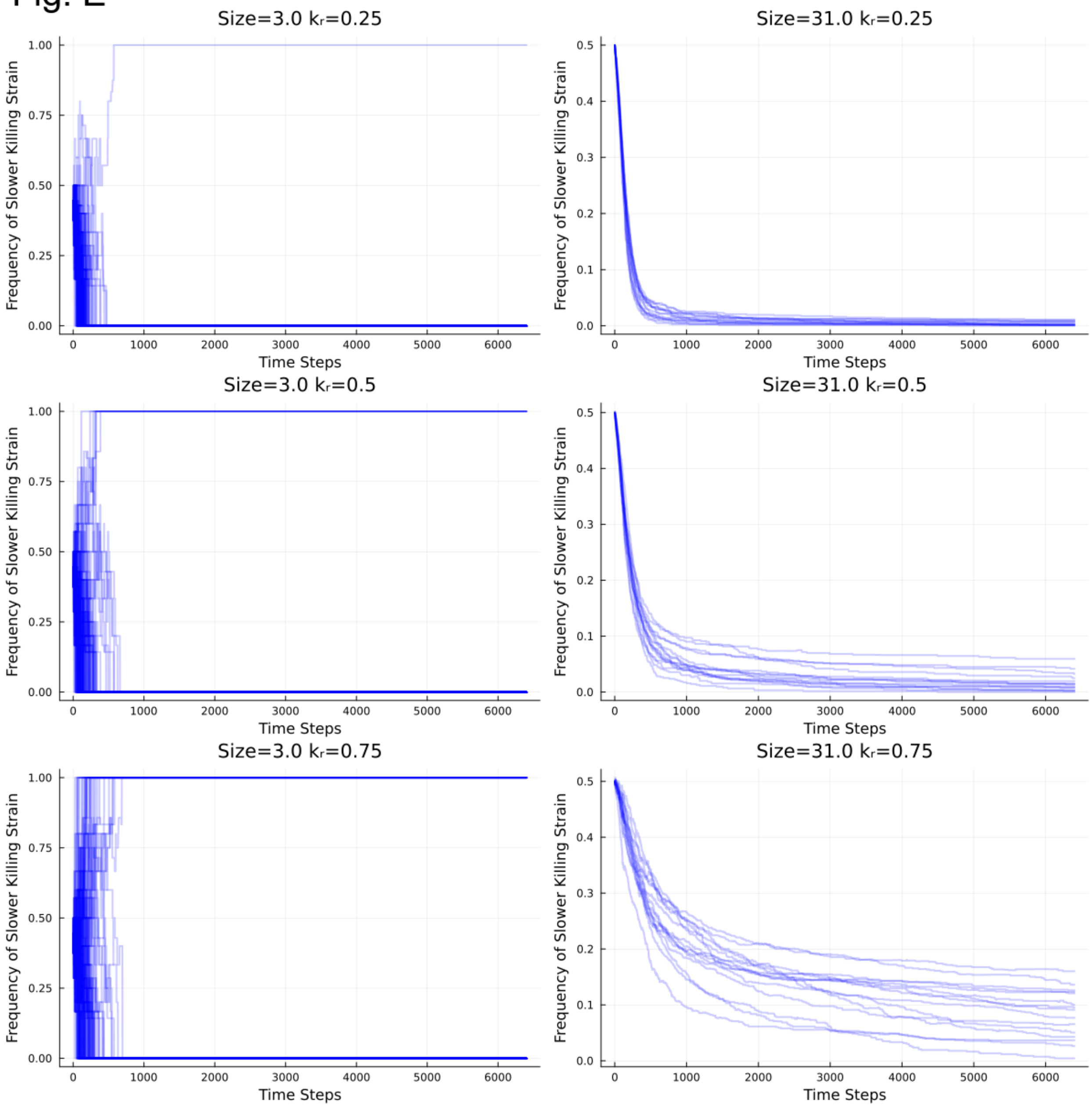

Fig. E The frequency of the slow killing strain is plotted versus time steps; every 100 time steps is a generation as this is the average time taken for one cell to divide. All panels show 15 trends. Size 3 and 31 correspond to 9 and 1025 cell simulations respectively.

Next, we show histograms of the final frequency of each strain. These histograms show that the small populations end with a slow killing abundance of 0 or 1, i.e., all or nothing, while the large populations have a much narrower spread of final abundances, resulting in a small variance.

Fig. H

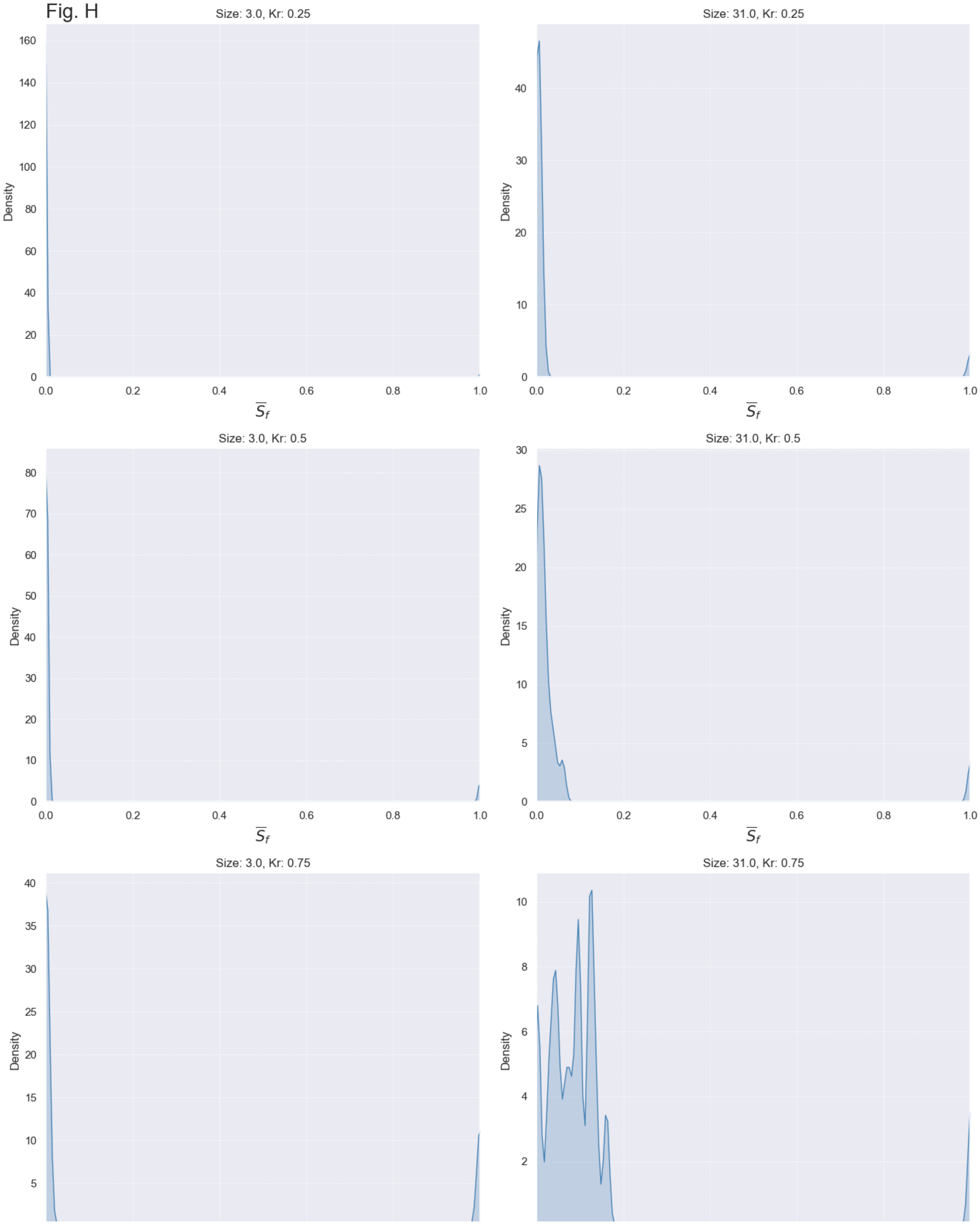

Size: 3.0, Kr: 0.25
Size: 31.0, Kr: 0.25
Size: 3.0, Kr: 0.5
Size: 31.0, Kr: 0.5
Size: 3.0, Kr: 0.75
Size: 31.0, Kr: 0.75
Density
$\overline{S}_f$

Fig. H Here we show the probability density functions of the final proportions of the slow killing strain taken from the frequency over time examples shown above.

Overall, the research done is interesting but I think that clarifying the points above should significantly strengthen the manuscript and its conclusions.

Some other minor comments:

On page 8, line 175, the authors state that 'there are more initial conditions that enable 175 the survival of the slow killer in the small system than there are in the large system.' This statement, if I understand correctly, refers to the fact that the region above the dashed white line in Fig. 3A is larger than the region above the red dashed line in Fig. 3C. It seems that the conclusion here would be the same for small values of \bar S_f, but interestingly it seems as if this is not true for larger values, e.g. \bar S_f = 0.5. Although I do understand that for this statement a small value of \bar S_f is appropriate, I thought it would be interesting to compare the relative extent of the regions of parameter space in which \bar S_f > 0.5 between small and large populations. I would also recommend using the same axes ranges for the four panels, as it would facilitate comparison across panels.

Response: Thank you for this suggestion. In fig 6, we explore more robustly the parameter space that ends in either strain increasing their relative frequency; we found that stochasticity in small systems helps whoever loses in the large system. Further, Fig 3 A's axis range comes from the small N present in 9 cell colonies. In response to your comment and others, we have removed the language comparing the size of region space in Fig 3.

The title of Fig. 4 could be more precise by saying 'Sampling fluctuations of initial conditions in small systems favor the slow killing strain'

Response: Thank you for this suggestion; we agree and have changed the title to "Sampling fluctuations of initial conditions in small systems favor the slow killing strain"

At the end of page 8, line 181, does 'these fluctuations' refer to those in Fig. 2, since in Fig. 3 the initial conditions were deterministic? The location of this sentence within the text suggests the opposite, it might be worth specifying which fluctuations are being referred to.

Response: Thank you for pointing this out. We agree, this sentence was not clear. We edited it to now say "To understand the source of fluctuations in stochastically seeded simulations

\ref{FIG-2}, we next delineated the distribution of initial conditions."

Line 192, 'platonic' should read 'planctonic'

Response: Thank you for catching this typo; we have corrected it.

Do the authors see any significant difference between Fig. 5 and Fig. 3? I thought a comparison would be warranted.

Response: We completely agree. We added graphs to figures 2 and 5 to explore the difference between stochastic and deterministic seeding. Specifically, Figs. A and B from this response document (described in response to your questions above) are now included in Fig. 2 of the main text, and Fig 5 B now shows the frequency of the slow killing strain with deterministic initial conditions. We find that, in line with our response to your comment that starts with "On the theoretical side", both stochastic seeding and small colony sizes aid the slow killing strain in survival. In particular, stochastic seeding yields higher frequencies as k_r decreases.

The back-and-forth between sampling and non-sampling the S_i distribution is at times a bit confusing and reader is left wondering which of the two are being discussed. It would help more clearly identify which figure
refers to which situation, or discussing deterministic S_i first in the text, and then the sampling

Response: We agree. We edited the language and added graphs to this end.

Lines 230-231: here it would be helpful to have a comparison with deterministic, fixed initial conditions.

Response: Thank you for pointing this out; we agree. The changes made to this end are described above in response to your comment that starts "Do the authors see any…".
